# Valley-Dependent Emission Patterns Enabled by Plasmonic Nanoantennas

**DOI:** 10.1021/acsnano.5c11672

**Published:** 2026-03-24

**Authors:** Tobias Bucher, Jingshi Yan, Jan Sperrhake, Zlata Fedorova, Mostafa Abasifard, Rajeshkumar Mupparapu, Haitao Chen, Emad Najafidehaghani, Khosro Zangeneh Kamali, Antony George, Mohsen Rahmani, Thomas Pertsch, Andrey Turchanin, Dragomir N. Neshev, Isabelle Staude

**Affiliations:** † Institute of Solid-State Physics, 9378 Friedrich Schiller University Jena, Jena 07743, Germany; ‡ Institute of Applied Physics, 9378 Friedrich Schiller University Jena, Jena 07745, Germany; § Abbe Center of Photonics, 9378 Friedrich Schiller University Jena, Jena 07745, Germany; ∥ ARC Centre of Excellence for Transformative Meta-Optical Systems (TMOS), Research School of Physics, 2219 Australian National University, Canberra 2601, ACT, Australia; ⊥ Institute of Physical Chemistry, 9378 Friedrich Schiller University Jena, Jena 07743, Germany; # Fraunhofer Institute for Applied Optics and Precision Engineering IOF, Jena 07745, Germany; ¶ Max Planck School of Photonics, Jena 07745, Germany; ∇ Jena Center for Soft Matter (JCSM), Jena 07743, Germany

**Keywords:** valley-momentum coupling, directional emission, monolayer TMDs, valleytronics, plasmonics, nanoantennas

## Abstract

Selective control of the emission pattern of valley-polarized excitons in monolayer transition metal dichalcogenides is essential for advancing valleytronic, quantum information, and optoelectronic devices. Although substantial progress has been made in directionally routing photoluminescence from these materials, key challenges persist: specifically, establishing how observed routing effects relate to the degree of valley polarization and distinguishing genuine valley-dependent routing from spin-momentum coupling, an optical scattering effect unrelated to the emitter. In this work, we address these challenges by experimentally and numerically demonstrating a direct link between excitonic valley polarization and the resulting farfield emission pattern, enabling quantitative evaluation of valley-selective emission routing. We report valley-dependent manipulation of the angular emission pattern of monolayer tungsten diselenide using gold nanobar dimer antennas at cryogenic temperatures. By probing the emission under opposite circularly polarized excitation, we observe a valley-selective asymmetry in the photoluminescence circular dichroism of 2%. These measurements are supported by a reciprocity-based numerical framework that enables modeling of valley-selective emission in periodic systems. Our calculations further reveal that the observed valley-dependent directionality is a symmetry-protected property of the nanoantenna array arising from its extrinsic chirality at oblique emission angles, and that it can be substantially enhanced by tailoring the emitter distribution. Together, these results establish our nanoantenna platform as a robust route toward valleytronic signal processing.

## Graphical abstract

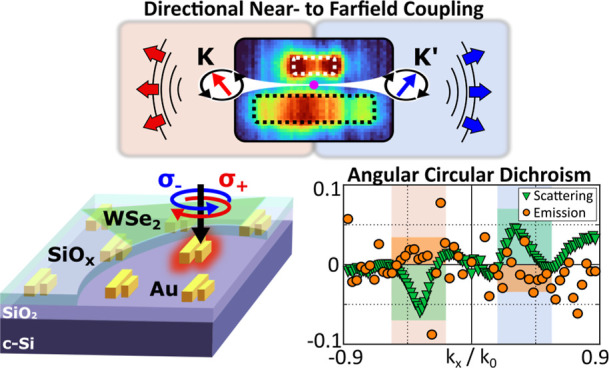

Two-dimensional semiconducting transition metal dichalcogenides (2D-TMDs) possess unique optoelectronic properties, which have propelled them in the spotlight of research in photonics and material science during more than a decade.^1–4^ Among them are a strong direct-bandgap photoluminescence (PL)^5,6^ and a high second-order nonlinear susceptibility in the monolayer phase,^7,8^ as well as a pronounced excitonic response at room temperature.^9,10^ Furthermore, the valley pseudospin in 2D-TMDs introduces a new binary degree of freedom for electrons that may be utilized to encode information, paving the way for novel approaches in information processing and storaging.^11–21^ The valley pseudospin arises from multiple energetically degenerate but spin-selective band extrema, the so-called valleys, in the conduction and valence bands of a crystal. These valleys form at the direct bandgaps located at the corners of the Brillouin zone, where carriers occupy one of the two subsets (K or K′ valleys) depending on their spin state. Additionally, the optical selection rules become valley-dependent, allowing spin-polarized valleys to be selectively addressed and read out using circularly polarized light.^22,23^ The degree of valley polarization (DOVP) reflects the contrast between exciton densities in different valleys and is typically measured through the circular polarization of the emitted PL. While the instantaneous DOVP can reach values close to ±1, strong intervalley electron-hole exchange interaction leads to a fast valley depolarization, thus limiting the time available for logical processing, transporting and detecting the valley information even at cryogenic temperatures.^24–26^

Photonic nanostructures offer intriguing opportunities for interfacing 2D-TMDs with light at the nanoscale.^27–29^ In particular, they have proven their potential to contribute to solutions for reducing the valley depolarization via various mechanisms. One strategy is to enhance the circular polarization contrast by using chiral nanoparticles,^30^ chiral assemblies of metallic nanoparticles,^31^ chiral^32–34^ or achiral^35^ metasurfaces, and other tailored designs.^29^ Another approach uses engineered nanostructures to achieve valley-selective directional coupling of valley-polarized excitons or of their emitted light.^14,18,36–41^

However, most experimentally studied structures for valley routing–whether based on propagating surface plasmon polaritons^38^ and guided modes,^14,18,36^ or extended modes in metasurfaces^37,39^ and photonic crystals^40–42^ –typically have large footprints of several square microns. The large size of the suggested structures is problematic considering the high integration densities that would be ultimately required for valleytronic devices. A solution to this problem is provided by plasmonic nanoantennas, which are well-known for their ability to shape the emission patterns of localized sources.^31,43–46^ Plasmonic nanoantennas have also been demonstrated to facilitate free-space emission routing for rotating electric dipole sources, scattering light into different angular directions depending on the rotation direction of the nearfield source.^31,47^ Importantly, this approach directly applies to the concept of valley routing, as rotating electric dipoles accurately model the emission from valley-polarized excitons in 2D-TMDs.^14,35,37,39,48,49^

An ideal valley-routing device should scatter PL from valley-polarized excitons into distinct directions based on the dipole’s rotation direction, while preserving circular polarization in the farfield to faithfully reflect the underlying DOVP. A natural approach to assess such functionality is through angle- and polarization-resolved measurements of the emitted light. However, interpreting these measurements can be misleading due to the interplay between spin- and valley-dependent effects. Spin-momentum coupling, for instance, can produce directional emission patterns linked to circular polarization regardless of the emitter’s internal valley state.^45,46^ As a result, such routing effects do not reliably indicate the DOVP. Additionally, the scattering process itself can alter the polarization state of the emitted light in a complex manner,^48,50,51^ further complicating interpretation. In this study, we address these challenges by establishing a direct connection between the intrinsic DOVP of the material and the farfield emission characteristics, enabling an accurate assessment of valley-selective emission routing.

This connection is established through combined experimental and numerical analyses of valley-selective directional emission from monolayer tungsten diselenide (1L-WSe_2_) integrated with a plasmonic nanoantenna array (Figure 1a). The nanoantennas consist of two parallel nanobars with different sizes, designed to support spectrally matched electric dipolar and electric quadrupolar resonances. Chen et al.^47^ numerically demonstrated that multipolar interference between these resonances leads to emission routing when both nanobars are simultaneously excited by a rotating electric dipole source, where the emission direction is linked to the dipole’s rotation sense.

**Figure 1:**
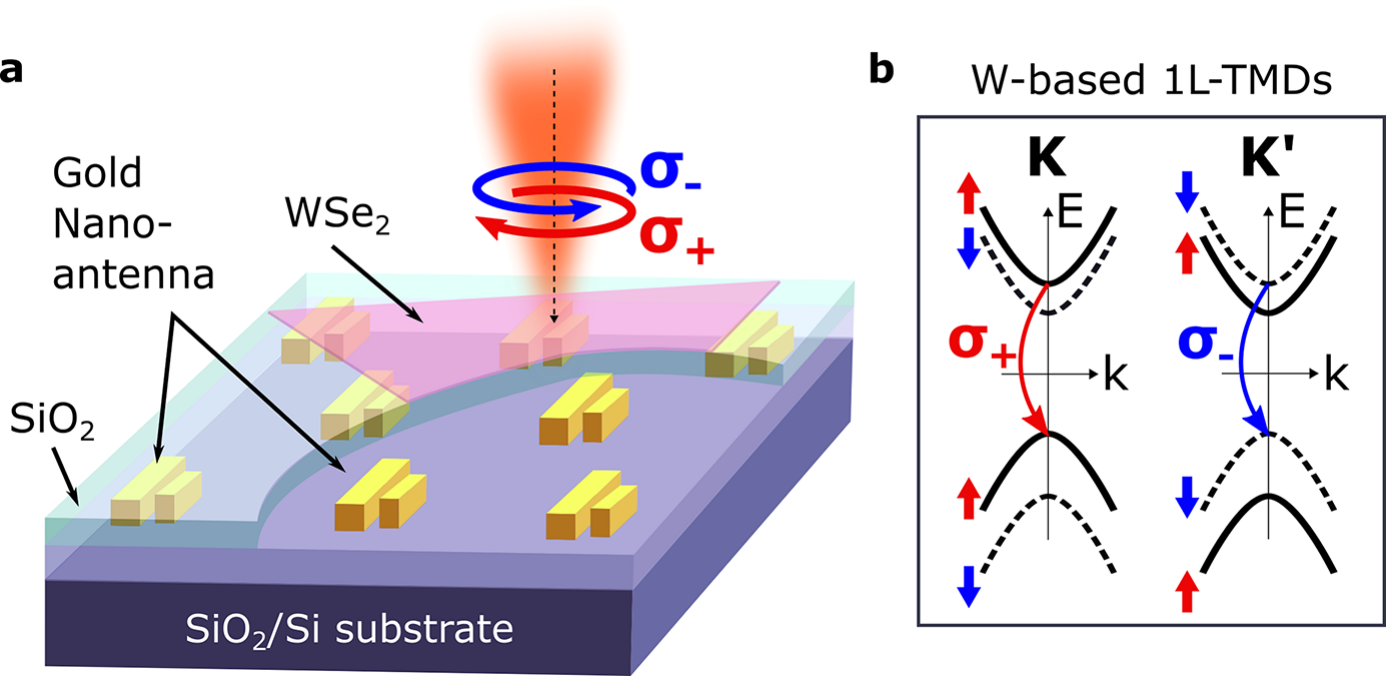
Nanoantenna concept for valley-routing. (a) Schematic of a hybrid system consisting of monolayer tungsten disulfide placed on top of an array of gold nanobar dimer antennas. In the monolayer, excitons can be selectively excited in specific valleys using circularly polarized light, as depicted in (b). Upon radiative decay, valley-excitons act as dipolar nearfield source for the plasmonic nanoantenna, facilitating directional multipolar interference and the corresponding valley-dependent emission directionality.

As a first step, we demonstrate that the subwavelength nanoantennas produce distinct angular scattering patterns when illuminated with circularly polarized white light of opposite handedness. We quantify this difference using a normalized contrast metric referred to as angular circular dichroism (CD). Unlike traditional CD measurements on chiral molecules or nanostructures, the chiral effects observed here are extrinsic, originating from directional scattering rather than structural chirality of the nanoantennas.

Next, we utilize the valley-dependent selection rules in 1L-WSe_2_ to generate a pronounced DOVP using circularly polarized excitation, as illustrated in Figure 1b. We then examine the nanoantenna-mediated directionality of the resulting PL, with particular focus on the angular CD obtained under both circularly polarized and unpolarized detection. By comparing these detection schemes, we are able to clearly distinguish purely electromagnetic scattering effects from those that genuinely reflect the underlying DOVP of the emitters.

We further support our experimental findings with numerical calculations of the angular CD in PL. To accurately model the emission of valley-polarized excitons embedded within the periodic nanoantenna array, we employe a reciprocity-based emission framework. Our simulations indicate that valley-selective directional scattering emerges from the interplay of multiple nearfield coupling mechanisms within the joint emitter-nanoantenna system. First, by systematically varying the lateral extent of the monolayer, we find that valley-polarized emitters exhibit larger angular CD when located closer to the nanoantennas. Second, we show that a robust directional response occurs along specific emission directions dictated by the extrinsic chirality of the asymmetric nanobar dimer, as no analogous directionality is observed in periodic arrays of single nanobars.

## Results and Discussion

### Nanoantenna Fabrication and Optical Characterization

Following the design proposed by Chen et al.,^47^ we fabricated hybrid structures of 1L-WSe_2_ transferred onto arrays of gold nanoantennas. The nanoantennas, each consisting of two parallel nanobars of different sizes, were patterned on an oxidized silicon substrate (300 nm silicon dioxide with an additional 10 nm indium tin oxide capping layer) using standard electron-beam lithography, gold evaporation and subsequent lift-off (see Methods for fabrication details). Figure 2a shows a scanning electron micrograph of a typical fabricated gold nanoantenna array, where the nanoantennas have a fixed height *H* = 40 nm and are arranged in a square lattice with a lattice constant Λ = 1 μm. Figure 2b shows a magnified view of the area indicated by the white box. The small and large nanobar have lateral dimensions of *l* × *w* = 100 nm × 40 nm and *L* × *W* = 300 nm × 65 nm, respectively, separated by a *g* = 50 nm gap. We fabricated additional arrays with systematically varied nanobar lengths *l* and *L*, to tune the dipolar and quadrupolar resonance wavelengths. To minimize changes in the carrier relaxation dynamics of the subsequently transferred 1L-WSe_2_, arising from charge-transfer, dipole-dipole interaction, or plasmonic quenching,^52,53^ we coated the nanoantenna arrays with a 15 nm silicon dioxide layer using physical vapor deposition.^54^

**Figure 2:**
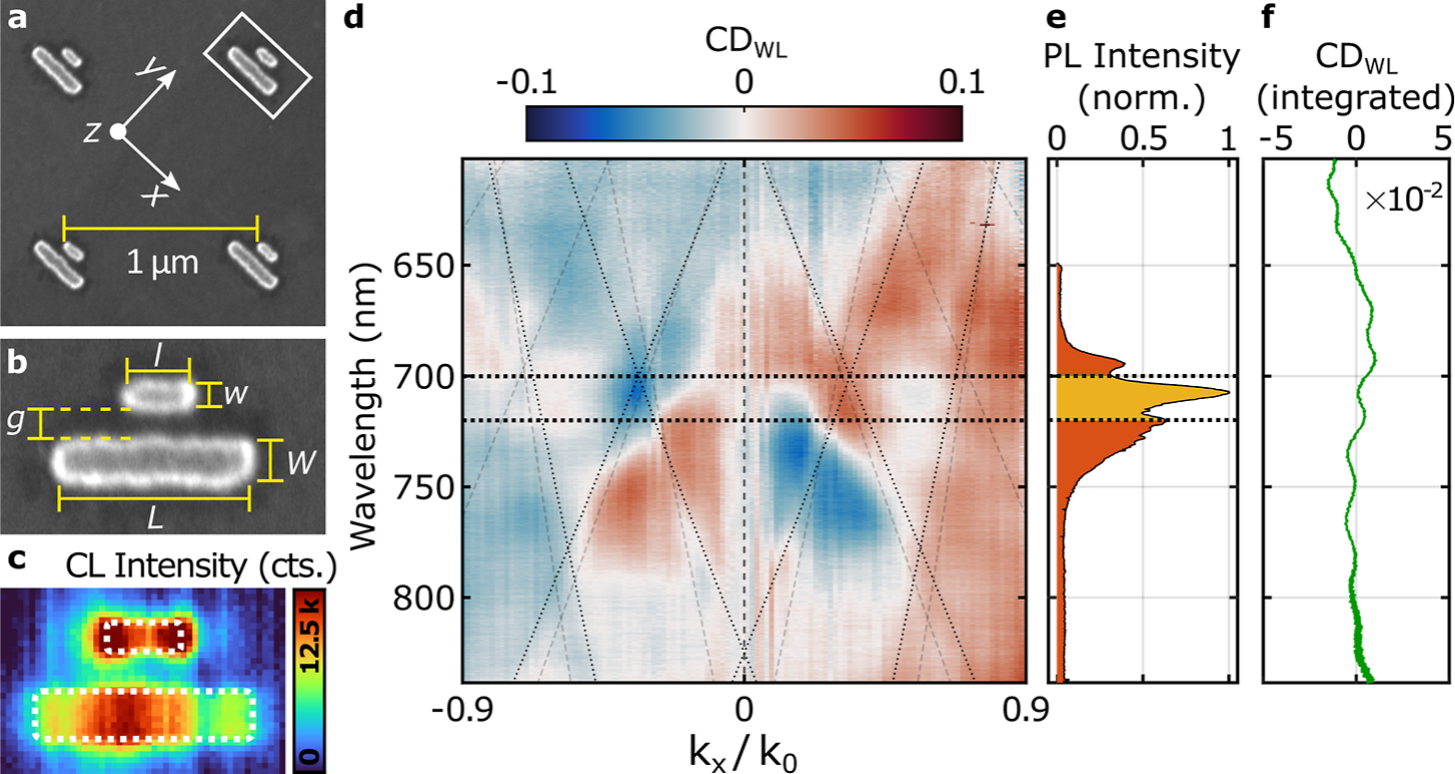
Optical characterization of the nanoantenna array. (a) Top-view scanning electron micrograph of a fabricated plasmonic double-bar nanoantenna array. (b) Close-up of a single nanoantenna as indicated by the white box in a) and definition of nanoantenna parameters. (c) CL scan of a nanoantenna from a similar array as shown above. The white dotted lines indicate the nanobar footprints. The CL signal corresponds to a wavelength range of 700–720 nm. (d) Measured angular CD_WL_ spectra retrieved from the backscattered light intensities $I_{WL}^{\sigma^{\pm }inc.}\left(k_{x},\lambda \right)$ upon illumination with σ ^±^ polarized light. The thin lines indicate the grating orders of the periodic nanoantenna array assuming refractive indices of *n*_1_ = 1.28 (dashed) and *n*_2_ = 1.65 (dotted). The thick dotted lines indicate the spectral range of detection of CL imaging.(e) Normalized cryo-PL spectrum of 1L-WSe_2_ on bare substrate. (f) Measured circular dichroism retrieved from the angle-integrated intensities ${Ĩ}_{WL}^{\sigma^{\pm }inc.}\left(\lambda \right)$ .

We characterized the optical nearfield response of the coated nanoantennas using cathodoluminescence (CL) imaging (see Methods for details). In this technique, a focused electron beam locally excites the nanostructure and induces photon emission through cascaded relaxation processes, providing access to optical nearfields with spatial resolution on the order of a few tens of nanometers.^55,56^ Figure 2c shows the corresponding CL map of a single nanoantenna from an array similar to the one depicted in Figure 2a, with the CL signal integrated over 700–720 nm, matching the expected trion emission band of 1L-WSe_2_ at cryogenic temperatures. The CL image reveals distinct nearfield mode profiles for the two nanobars. The smaller nanobar exhibits a hotspot at each end, characteristic of an electric dipole mode with dipole moment *p*_*x*_ oriented along its long axis. In contrast, the larger nanobar displays an additional hotspot near its center, consistent with a linear electric quadrupole mode with quadrupole moment *q*_*xx*_ aligned along the nanobar.

Simultaneous excitation of the dipolar and quadrupolar modes in the nanobars by a rotating dipole results in directional scattering, wherein the emitted light is preferentially routed into opposite halfspaces (*x* < 0 or *x* > 0) depending on the dipole’s spin orientation.^47^ In our system, the rotating dipole is provided by valley-selective excitonic emitters in 1L-WSe_2_, generated under circularly polarized optical excitation. We note that both nanobars are oriented along the *x*-axis (see Figure 2a).

Next, we investigated the circular-polarization-dependent directional scattering of the fabricated nanoantenna array using angle-resolved white light (WL) spectroscopy. To prepare circularly polarized illumination, we passed the WL output of a stabilized tungsten-halogen source through a linear polarizer (LP) followed by a superachromatic quarter wave plate (QWP). We illuminated the sample and collected the reflected light with a 100×/0.88 NA objective. By imaging the back-focal plane of the objective onto the slit of an imaging spectrometer, we then measured the angular spectra $I_{WL}^{\sigma^{\pm }inc.}\left(k_{x},\lambda \right)$ of the backscattered light under σ^±^ polarized illumination. Here, *k*_*x*_=(2π/λ) sin θ cos φ denotes the in-plane wave vector along the *x*-axis for spherical collection angles (θ, φ) ∈ [0, π/2] × {0, π}. A detailed discussion of the setup geometry and the influence of individual optical components on the excitation and detection polarization is provided in Section S.1 of the Supporting Information.

In Figure 2d, we show the corresponding angular CD defined as the normalized contrast CD_WL_ = (*I*_WL_^σ^+^inc.^ – *I*_WL_^σ^–^inc.^) / (*I*_WL_^σ^+^inc.^ + *I*_WL_^σ^–^inc.^). Between wavelengths of 700–720 nm (thick dotted lines), we observe a pronounced antisymmetric feature with respect to the two halfspaces *k*_*x*_ < 0 and *k*_*x*_ > 0, clearly showing circular-polarization-dependent directional scattering mediated by the nanoantenna array. In particular, we demonstrate a maximum CD of 6.5% at a wavelength of 705 nm, coinciding with the cryogenic PL peak wavelength of 1L-WSe_2_ on the bare substrate at 3.8 K, as shown in Figure 2e. We also observe several additional antisymmetric features between 700 and 800 nm. Because their dispersion closely follows that of the lattice modes (highlighted by the dashed lines), we attribute these signatures to an interplay between directional scattering from individual nanoantennas and the diffractive grating orders arising from their periodic arrangement. A detailed discussion of the grating order dispersion is provided in Section S.2 of the Supporting Information.

It is worth noting that both the individual nanoantennas and the nanoantenna arrays are geometrically achiral, such that the total CD of the array is expected to vanish. We verified this by evaluating the respective angle-integrated intensities ${Ĩ}_{WL}^{\sigma^{\pm }inc.}\left(\lambda \right)=\int_{NA}I_{WL}^{\sigma^{\pm }inc.}\left(k_{x},\lambda \right)dk_{x}$ . Figure 2f shows the resulting CD spectrum, which remains close to zero across the entire wavelength range (note the scale of 0.05). The small oscillatory deviations likely originate from the wavelength dependent birefringence of the polarization optics. We also emphasize that no polarization analysis was employed in detection. Thus, any nonzero CD solely reflects changes in the illumination polarization—a necessary condition for enabling valley-dependent routing.

Finally, we synthesized 1L-WSe_2_ on an oxidized silicon wafer via chemical vapor deposition using a Knudsen-type effusion cell, following the procedure described by George et al.^57^ This scalable method provides dense coverage of the growth substrate with single-crystalline monolayers, which we subsequently transferred onto the coated nanoantenna arrays using a poly­(methyl methacrylate)-assisted wet transfer scheme.^58^

### Photoluminescence Study of the Hybrid System

Figure 3a shows a true-color optical microscope image of a nanoantenna array after the monolayer transfer. In addition to monolayer regions, the crystal flake contains smaller areas of bilayer (2L) WSe_2_ and triangular holes, which appear with distinct optical contrast. We first investigated the PL emission of the hybrid system at room temperature without applying any polarization control. Figure 3b shows a confocal PL map of the same area (see Methods for experimental details). The 1L-WSe_2_ on the bare substrate exhibits bright and fairly uniform emission corresponding to the A-exciton PL. In contrast, the 2L-WSe_2_ regions appear darker owing to the significantly reduced quantum yield of the indirect-gap semiconductor. PL spectra of both 1L- and 2L-WSe_2_ are provided in Section S.3 of the Supporting Information. The PL signal from the triangular holes is indistinguishable from that of the bare substrate. Additionally, a regular square pattern of reduced PL intensity coincides with the positions of individual nanoantennas. This decrease in farfield PL intensity likely stems from the combined effects of scattering by the nanoantennas, diffractive grating modes of the array, and Fabry-Pérot resonances within the multilayer substrate. Interference among these contributions can modulate the detected PL intensity depending on the sample geometry and emission wavelength. For instance, we observe PL enhancement from 1L-WSe_2_ placed on nanoantenna arrays with shifted resonance wavelengths (see Section S.3 of the Supporting Information).

**Figure 3:**
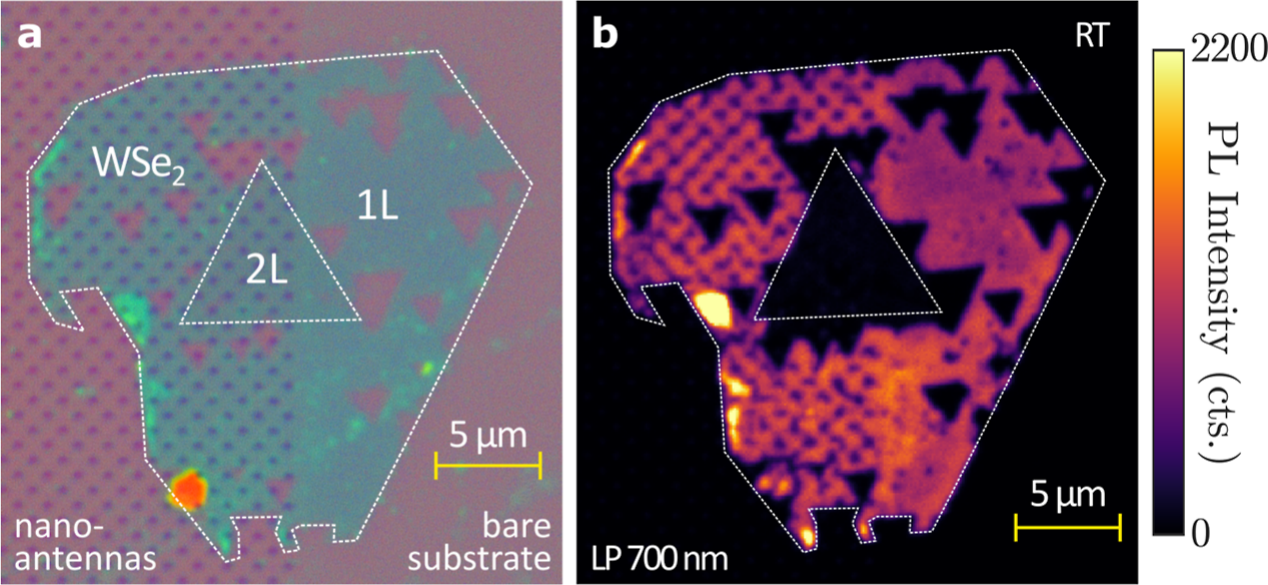
Room temperature measurements. (a) Optical microscope image of a 1L-WSe_2_ crystal transferred partly onto a fabricated gold nanoantenna array and partly onto bare substrate. The white dashed lines show the regions with the monolayer (1L) and bilayer (2L) WSe_2_, as labeled, respectively. (b) Measured confocal scanning microscope image of the PL from the same sample at room temperature using a 700 nm long-pass filter in detection. Room temperature PL measurements were conducted without employing circular polarization control of the excitation or detection.

We further investigated the influence of the resonant nanoantennas on the emission dynamics of 1L-WSe_2_ using time-resolved PL measurements (see Section S.3 of the Supporting Information). On the bare substrate, the PL exhibits a biexponential decay with lifetimes of (2.62 ± 0.05) ns and (0.37 ± 0.01) ns. For 1L-WSe_2_ on the nanoantenna array, these lifetimes decrease to (1.90 ± 0.06) ns and (0.32 ± 0.01) ns, indicating a measurable nearfield coupling between the excitons and the nanoantenna modes. Notably, this coupling is expected to strengthen at cryogenic temperatures, where the exciton resonance blueshifts into larger spectral overlap with the operational bandwidth of the nanoantenna.

Next, we investigated whether the fabricated nanoantenna arrays can directionally route the PL emission from valley-selective excitons in the 1L-WSe_2_. The induced valley contrast is typically characterized in emission by the degree of circular polarization (DOCP), defined as DOCP = (*I*_PL_^σ^+^det.^ – *I*_PL_^σ^–^det.^) / (*I*_PL_^σ^+^det.^ + *I*_PL_^σ^–^det.^). At room temperature, valley-selective excitation of 1L-WSe_2_ typically results in a negligible DOCP due to phonon-assisted ultrafast intervalley scattering. Cooling the sample to cryogenic temperatures substantially suppresses the intervalley scattering, allowing the circular polarization contrast of the excitation to be preserved at the emission level and resulting in a pronounced DOCP.

Crucially, upon radiative recombination, valley-selective excitons act as rotating dipolar nearfield sources that drive the nanoantenna at the exciton emission wavelength, with the dipole rotation following the circular polarization of the excitation.^48^ By design, the asymmetric nanobar dimer exhibits valley-selective directional farfield interference,^47^ thereby linking the emission direction to the excitonic valley-index and mediating valley-momentum coupling.

We performed circular-polarization resolved cryogenic measurements (*T* = 3.8 K) using a commercially available closed-cycle liquid-helium cryostat (s50, Montana Instruments) integrated into a custom-built back–focal plane imaging setup, as illustrated in Figure 4a. For excitation, we focused a 633 nm continuous-wave helium-neon laser with an average power of 100 μW on the sample using a 100×/0.88 NA objective, and collected the PL in reflection geometry with the same objective. For polarization control, we used a combination of LPs and QWPs. To compensate for the phase retardance introduced by the dichroic beam splitter (DBS), we placed an LP–QWP pair before the DBS to ensure a linearly polarized beam after reflection. After the DBS, another superachromatic QWP generated the σ^±^ polarized excitation before the beam entered the objective. In detection, the same superachromatic QWP converted the circularly polarized PL into a linear basis, which we analyzed with a second LP after reflection from a mirror (compare Section S.1 of the Supporting Information).

**Figure 4:**
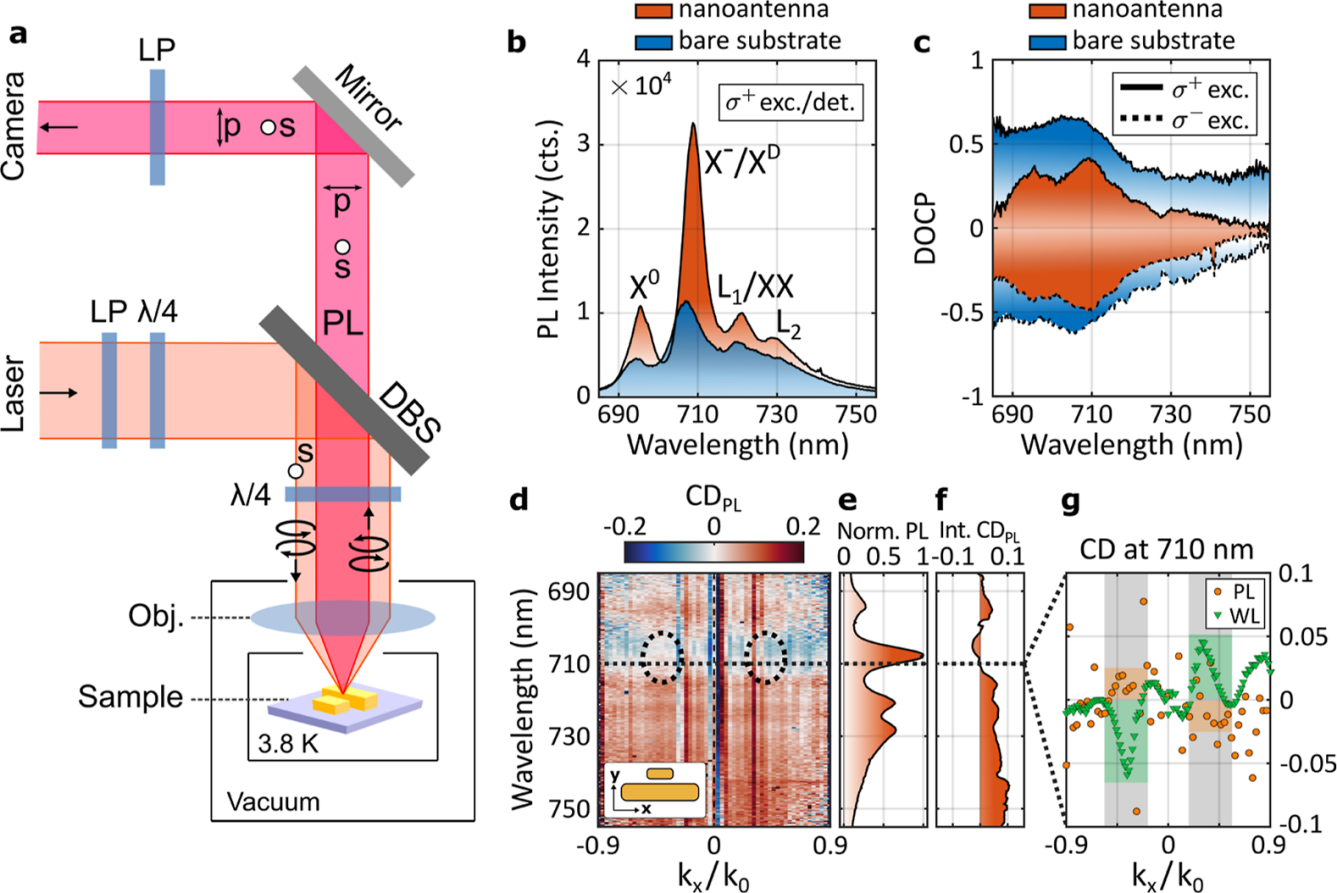
Cryogenic measurements (*T* = 3.8 K). (a) Sketch of the optical setup used for circular-polarization resolved cryogenic PL measurements, indicating the linear polarizers (LP) and quarter-wave plates (λ/4) placed before and after the dichroic beam splitter (DBS) for polarization control. (b) Intensity and (c) DOCP spectra of PL measured from 1L-WSe_2_ on the gold nanoantenna array (orange curve) and on the bare substrate (blue curve). (d) CD_PL_ as retrieved from the measured angular PL intensity spectra $I_{PL}^{\sigma^{\pm }inc.}\left(k_{x},\lambda \right)$ . (e) Integrated total PL intensity spectrum *I*_PL_(λ). (f) CD_PL_ as retrieved from the integrated PL intensity spectra $I_{PL}^{\sigma^{\pm }inc.}\left(\lambda \right)$ . (g) Cross section of the measured CD_PL_ (orange dots) and CD_WL_ (green triangles) at 710 nm wavelength. The dashed ellipses in (d) and the shaded areas in (g) were added as a guide to the eye, highlighting the angular range of interest.

Figure 4b shows PL spectra of 1L-WSe_2_ measured under σ^+^ polarized excitation and detection on the bare substrate (blue curve) and on the nanoantenna array (orange curve). In both cases, the spectra exhibit a characteristic multipeak structure typical of tungsten-based 1L-TMDs.^59–64^ In ascending wavelength order, the first peak corresponds to the neutral bright exciton (*X* ^0^). The second peak originates from several excitonic complexes, including negatively charged trions (*X* ^–^) as well as spin-forbidden dark excitons (*D* ^0^) and trions (*D* ^–^), and is therefore labeled *X* ^–^/*D*. Two additional peaks at longer wavelengths are attributed to localized defect states, with the third peak also spectrally overlapping the expected emission energy of the biexciton (*XX*). Accordingly, we label the third and fourth peak as *L*_1_/*XX* and *L*_2_, respectively. A detailed decomposition of the excitonic contributions based on pseudo-Voigt line-shape fitting is provided in Section S.4 of the Supporting Information.

On the nanoantenna array, the PL intensity is enhanced relative to the bare substrate across the entire emission spectrum of 1L-WSe_2_, consistent with excitation and emission enhancement mediated by the nanoantennas (see Section S.3 of the Supporting Information for complementary time-resolved measurements at room temperature). In addition, the PL spectrum of 1L-WSe_2_ on the nanoantennas exhibits a redshift by several meV. Although such spectral shifts can arise from spatial variations within the monolayer (compare Figure S4b), we observe a larger shift for the *X* ^–^/*D* peak, suggesting a possible nearfield-induced brightening of dark excitons (compare Section S.4 of the Supporting Information).

Next, we measured the DOCP of the PL emission from 1L-WSe_2_, as shown in Figure 4c, for regions on the bare substrate (blue curve) and on the nanoantenna array (orange curve) under σ^+^ (solid curves) and σ^–^ (dashed curves) polarized excitation. In both cases, the highest DOCP is associated with the *X* ^–^/*D* peak, reaching values of 0.65 for the bare substrate and 0.43 on the nanoantenna array. The reduced DOCP observed for 1L-WSe_2_ on the nanoatenna array occurs across the entire emission spectrum and is therefore attributed to polarization-altering scattering by the plasmonic nanoantennas,^48^ as further corroborated by numerical emission modeling in Section S.9 of the Supporting Information. We note that the slight asymmetry observed in the DOCP spectrum on the nanoantenna array is not expected for an achiral structure and likely originates from small differences in the probed sample position between measurements performed with different excitation polarization.

To quantify the valley dependence of the emission patterns, we performed angle-resolved spectroscopy of the PL emitted by 1L-WSe_2_ placed on the gold nanoantenna array. We employed the same detection scheme as in the angle-resolved white light spectroscopy, while expanding the excitation beam to illuminate the same sample area as used for the focused white light measurements (see Methods and Section S.1 of the Supporting Information). Figure 4d shows the corresponding angular CD in PL, defined as CD_PL_ = (*I*_PL_^σ^+^exc.^ – *I*_PL_^σ^–^exc.^) / (*I*_PL_^σ^+^exc.^ + *I*_PL_^σ^–^exc.^), which we retrieved from the angular PL spectra $I_{PL}^{\sigma^{\pm }exc.}\left(k_{x},\lambda \right)$ . For reference, Figures 4e,f show the total PL intensity spectrum *Ĩ*_PL_ = *Ĩ*_PL_^σ^+^exc.^ +*Ĩ*_PL_^σ^–^exc.^ with ${Ĩ}_{PL}^{\sigma^{\pm }exc.}\left(\lambda \right)=\int_{NA}I_{PL}^{\sigma^{\pm }exc.}\left(k_{x},\lambda \right)d{\;k}_{x}$ , and the corresponding CD_PL_ calculated from these angle-integrated spectra. In contrast to the white-light scattering measurements, the CD_PL_ does not exhibit a pronounced directional pattern. Instead, we observe only a weak antisymmetric feature near the spectral position of the maximum DOCP, as indicated by the dotted line and circles. For clarity, Figure 4g shows a cross-section of CD_PL_ at a wavelength of 710 nm (orange dots), alongside the corresponding cross-section of CD_WL_ measured on the nanoantenna array without the monolayer (green triangles). Whereas CD_WL_ displays a pronounced antisymmetric angular dependence, the CD_PL_ distribution appears less structured. Nevertheless, within the angular range discussed previously and highlighted by the shaded regions, we identify a measurable antisymmetric component in CD_PL_ with a magnitude of approximately 2%, whose sign is opposite to that observed in the white light measurements. Note that, although the integrated CD_PL_ must vanish for an achiral nanoantenna array, we observe subtle spectral modulations over the course of successive measurements. In particular, the *X* ^–^/*X* ^*D*^ peak near 709 nm diminishes, while the *X* ^0^ peak and a broad defect band increase in intensity. We attribute these changes to slow, photoinduced charge effects,^65^ occurring on a time scale comparable to the interval between consecutive measurements. Importantly, these spectral variations do not influence the directional CD_PL_ at any fixed wavelength.

### Valley-Selective Directional Scattering

Next, to gain deeper insight into the observed valley-selective directional asymmetry, we performed polarization-resolved back-focal plane imaging (see Methods for details). Figure 5a shows the angular PL intensity acquired at the same position on the nanoantenna array discussed above using an additional (710 ± 5) nm bandpass filter. We specifically examined changes in the angular PL distribution upon switching the circular polarization of the excitation (top and bottom rows) and analyzed the resulting emission patterns using circular-polarized detection channels (left and middle column), as well as unpolarized detection (right column). For clarity, we adapt a Jones notation to distinguish the polarization configurations, defining, for example, σ_det._^+^|σ_exc._^–^≡+–, with analogous notation used for all other polarization combinations. The resulting emission patterns exhibit pronounced arc-like features arising from diffractive grating orders associated with the periodic nanoantenna array, as discussed in Section S.2 of the Supporting Information. For comparison, the calculated diffraction patterns are overlaid in the bottom-left panel of Figure 5a.

**Figure 5:**
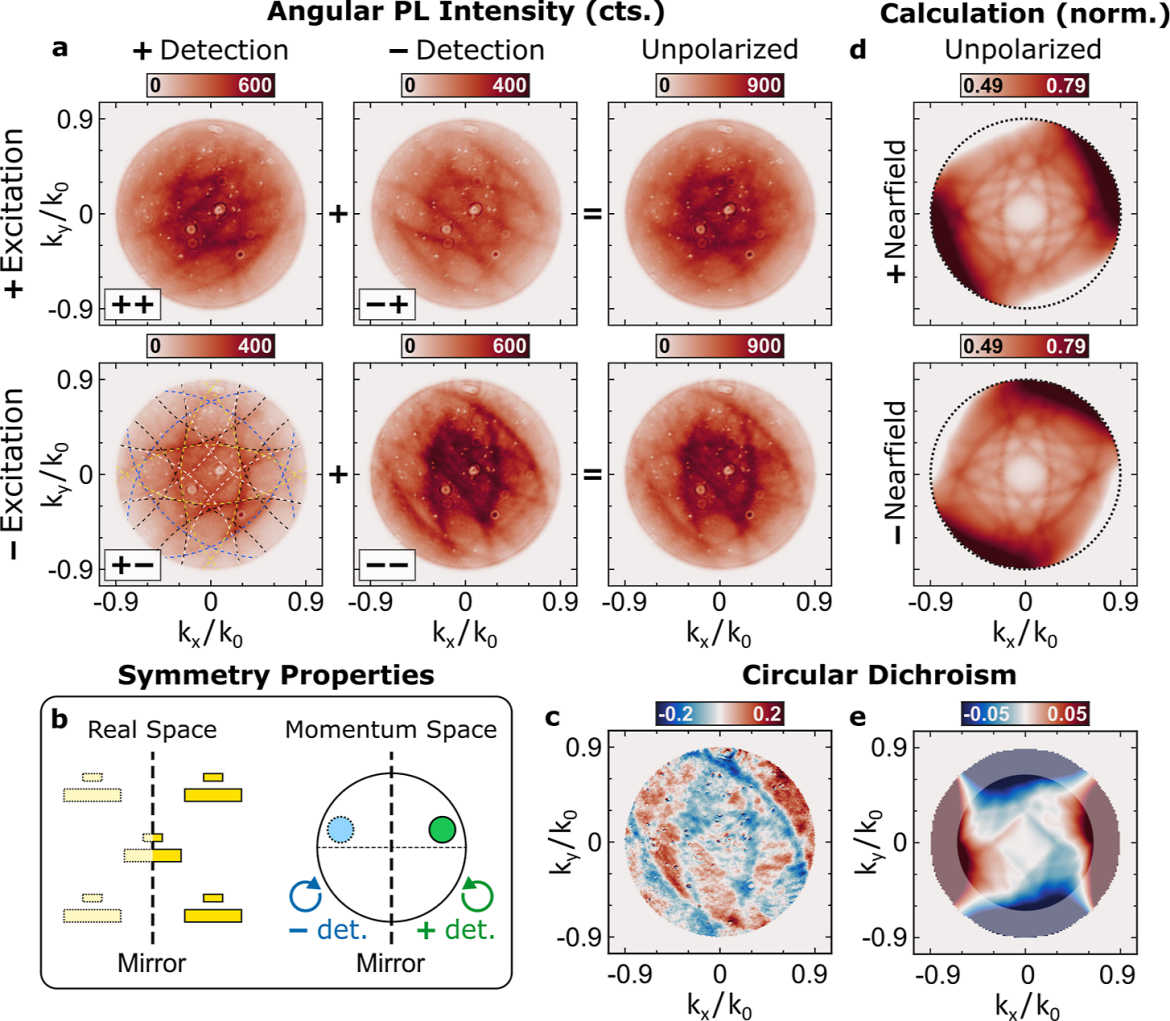
Angular PL intensity distribution and circular dichroism. (a) Measured angular PL intensity distribution of 1L-WSe_2_ on the gold nanoantenna array for σ^+^ (top row) and σ^–^ (bottom row) polarized excitation and σ^+^ (left column), σ^–^ (middle column), and unpolarized (right column) detection. The dashed lines in the bottom left indicate the grating orders for a periodic nanoantenna array assuming a refractive index of *n*_1_ = 1.28. (b) Sketch of the system’s symmetry properties in real and momentum space. (c) Measured angular circular dichroism, obtained from the unpolarized case in (a). (d) Numerically calculated angular emission intensity distributions obtained from σ^+^ (top) and σ^–^ (bottom) polarized nearfield intensities, averaged over circular farfield polarizations according to the reciprocity-based approach. (e) Numerically calculated angular circular dichroism, obtained from (d).

In these emission patterns, the diffractive modes do not appear with uniform intensity. Instead, preferred emission directions emerge depending on the circular polarization of detection. Specifically, for the––and–+ configurations (i.e., σ^–^ polarized detection), the diffractive modes are enhanced along diagonal directions in momentum space (*k*_*x*_·*k*_*y*_ > 0), whereas for the ++ and +–configurations (i.e., σ^+^ polarized detection), emission is favored along the antidiagonal directions (*k*_*x*_·*k*_*y*_ < 0). This pronounced polarization dependence constitutes a clear signature of spin-momentum coupling mediated by the nanoantenna array. Moreover, these emission patterns are related by the symmetry of the system in momentum space, as illustrated in Figure 5b. The patterns corresponding to opposite detection polarizations appear as mirror images with respect to the *k*_*y*_-axis, as a consequence of the mirror symmetry of the nanoantenna array and the associated reversal of handedness induced by the mirror operation. Notably, this symmetry is partially lifted when valley-selective excitation is introduced. For a fixed detection polarization, changing the excitation polarization modifies the emission intensity but not the overall shape of the angular pattern, consistent with the finite DOCP associated with valley-polarized exciton emission.

These observations demonstrate that the angular emission distribution in our system is sensitive to the valley polarization. For unpolarized detection (right column in Figure 5a), this polarization dependence manifests as clearly distinct emission patterns. The corresponding angular CD map shown in Figure 5c provides a quantitative measure of these differences, revealing pronounced contrast across multiple emission directions. We find angular CD magnitudes of up to 15% for selected emission directions, as well as antisymmetric features reaching amplitudes of up to ±6% along the *k*_*x*_ axis. For comparison with the results presented in Figure 4g, see Section S.5 of the Supporting Information. Importantly, the finite angular CD constitutes a direct signature of valley-selective emission with respect to propagation direction. By combining valley-selective excitation and unpolarized detection, we isolate directional effects that arise purely from valley-momentum coupling, thereby distinguishing our results from earlier demonstrations of spin-momentum locked emission observed in systems without an intrinsic valley degree of freedom^45^ or lacking valley-selective excitation.^46^

We further performed numerical simulations of the emission from the studied system using a reciprocity-based model.^66–68^ This approach exploits the equivalence, established by Lorentz reciprocity, between the farfield emission of a single dipolar source placed in the nearfield of a nanophotonic structure and the nearfield response of the same structure under farfield illumination. A detailed description of the numerical implementation is provided in Methods. The model enables calculation of the radiated power and farfield polarization of incoherent dipolar emitters embedded in periodic systems. To simulate the emission from 1L-WSe_2_ on the nanoantenna array, we independently calculated the in-plane nearfield distributions at the surface of the embedding layer under illumination with two orthogonally polarized plane waves.^66^ Within this framework, valley-selective emitters in the K/K′ valleys are associated with the σ^+^ and σ^–^ polarized components of the in-plane nearfield, and the polarization of the emitted light is assigned to that of the incident plane waves driving the system.

By varying the angle of incidence, we then calculated the angular emission intensity distributions for unpolarized detection, obtained by summing over both orthogonal farfield polarization states. The resulting distributions are shown in Figure 5d for the σ^+^ polarized (top) and σ^–^ polarized (bottom) nearfield components, together with the corresponding angular CD shown in Figure 5e. The simulations reproduce diffractive mode patterns analogous to those observed experimentally, giving rise to similar symmetry-related features discussed above. The calculated emission patterns for circularly polarized detection are provided in Section S.6 of the Supporting Information. Importantly, the simulated angular CD distribution corroborates the magnitude of the experimentally observed left–right asymmetry for nanoantenna arrays homogeneously covered by 1L-WSe_2_. In particular, for small emission angles, we obtain antisymmetric features with angular CD values up to ±1%. We therefore conclude that the valley selectivity in the experimentally measured angular CD maps is primarily reflected in the left–right asymmetry (up to ∼2%), rather than in the local extrema observed at specific emission angles (up to ∼15%), as discussed in detail in Section S.5 of the Supporting Information. The slightly underestimated angular CD asymmetry in the simulations compared to the experiments is attributed to additional excitation enhancement mediated by the resonant nanoantennas, which is not included in our approximate numerical framework. At larger emission angles, the model tends to overestimate the emission intensities and, consequently, the contrast values, as such pronounced features are not observed experimentally. In addition to excitation-related effects, we attribute this discrepancy to limitations of the model at grazing emission angles, arising from the projection of the circularly polarized emission states onto the plane of the monolayer.

### Emitter Distribution

We recall that both the experimentally measured and numerically calculated angular CD asymmetries remain systematically below those predicted for valley-selective emitters located in the immediate vicinity of an isolated nanoantenna.^47^ This discrepancy likely arises from contributions of emitters located farther from the nanoantenna, experiencing weaker nearfield interactions. To test this hypothesis, we systematically varied the lateral extent of the monolayer in the numerical simulations, thereby gradually reducing the contributions of these remote emitters. In this analysis, we treated the monolayer coverage within a single unit cell as a free design parameter while keeping all other structural parameters fixed.

Exemplary, we consider three representative cases (A–C) in which monolayers of different lateral extent are placed within a single unit cell of the periodic array, as illustrated in Figure 6a. The corresponding calculated angular CD distributions for these cases are shown in Figure 6b. For case A, where the monolayer is confined to the gap region between the nanobars, the angular CD magnitude reaches values up to 53%. As the monolayer area is gradually increased (cases B and C), the magnitude of the angular CD decreases substantially, owing to the dominant contribution of emitters that are weakly coupled to the nanoantenna and therefore do not exhibit a valley-selective directional contrast. This increasing contribution of uncoupled emitters also leads to a qualitative change in the angular CD distribution, as is evident when comparing cases A and C. In case A, the pattern predominantly reflects the nanoantenna’s antisymmetry with respect to the *k*_*y*_ axis (see also Figure 5b), consistent with efficient nearfield interaction of emitters located within the nanoantenna gap. In contrast, for case C, where most emitters are located farther from the nanoantenna, the angular CD exhibits a 2-fold antisymmetric distribution that originates from the square lattice symmetry of the array.

**Figure 6:**
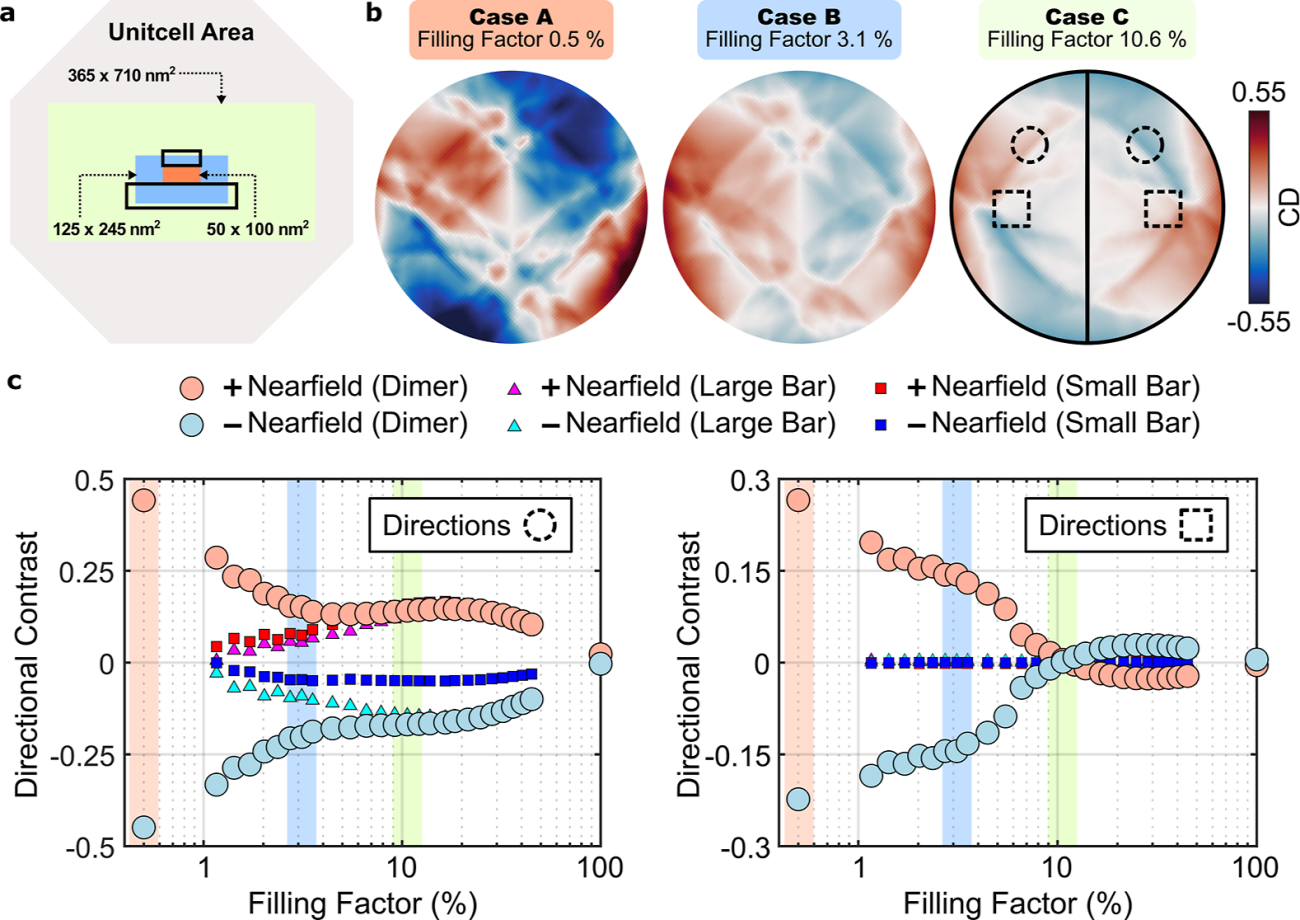
Effect of the emitter distribution. (a) Top-view sketch of differently sized monolayers (orange, blue, and green) covering the unitcell area (gray) of the nanoantenna array. (b) Numerically calculated angular circular dichroism of the nanoantenna array for different monolayer areas. Here, the spatial directions align with the nanoantenna sketch shown in (a) and the angular range coincides with the experimental NA of 0.9. (c) Retrieved directional intensity contrast as a function of the monolayer filling factor obtained for the directions indicated by the dashed circles (left) and dashed squares (right). Valley-selective results are obtained respectively from σ^+^ and σ^–^ polarized nearfield components for the nanoantenna array (red and blue circles), as well as for arrays of the individual large nanobar (magenta and cyan triangles) and the small nanobar (red and blue squares).

Based on these considerations, we introduce a quantitative metric to characterize the strength of the valley-routing effect. We consider two farfield detectors that measure the intensities *I*_left_ and *I*_right_ emitted into two fixed propagation directions within the left and right halfspace, respectively. As an initial choice, we selected the two directions indicated by the dashed circles in Figure 6b. We then define a measurable directional contrast (*I*_left_ – *I*_right_)/(*I*_left_ + *I*_right_), which quantifies the asymmetry of the emission between the two directions. The left panel of Figure 6c shows this directional intensity contrast as a function of the monolayer filling factor within a single unit cell of the nanoantenna array (round markers). For a fixed valley state, represented by the circularly polarized nearfield components (red and blue markers), this contrast yields finite values, with larger contrasts obtained for smaller filling factors. Importantly, the sign of the contrast follows the valley index, enabling an efficient farfield readout of the valley information even in the presence of a resonant nanoscatterer, which may otherwise obscure this correspondence through its scattering response.^48^

For comparison, we also calculated the directional intensity contrast for arrays of individual nanobars, using the same geometric parameters as the small nanobar (red and blue squares) and the large nanobar (magenta and cyan triangles). For small filling factors, these monomer arrays exhibit markedly different directional scattering behavior compared to the nanobar dimer array. We therefore attribute the large contrast values observed for filling factors below ∼3% to the resonant interaction between the two nanobars forming the dimer. For larger filling factors, the contrast differences between dimer and monomer arrays become negligible, as the directional scattering is increasingly dominated by diffractive lattice modes.

Alternatively, the right panel in Figure 6c shows the directional contrast evaluated for a different pair of emission directions, indicated by the dashed squares in Figure 6b. In this case, a clear distinction between the nanobar dimer array and the monomer arrays persists across the entire range of filling factors. This behavior indicates that, for this choice of directions, the measured contrast arises exclusively from valley-selective directional scattering mediated by the nanoantenna, without contributions from diffractive modes.

We note that these two cases highlight the presence of two distinct symmetries in the system: the lattice symmetry of the periodic array and the intrinsic symmetry of the dimer nanoantenna. Crucially, only for the dimer nanoantenna does the left–right directional intensity contrast constitute a symmetry-protected property, enabled by its extrinsically induced chirality.^69–72^ A detailed symmetry analysis is provided in Section S.7 of the Supporting Information. Consequently, when integrating over all farfield directions within each halfspace, the directional intensity contrast mediated by the nanoantenna dimer remains finite, whereas it vanishes for arrays composed of single nanobars. We have numerically verified this behavior, as discussed in Section S.8 of the Supporting Information. This symmetry-protected, valley-selective response therefore establishes the nanoantenna dimer as a robust platform for valleytronic processing, offering tolerance with respect to variations in farfield direction and emitter distribution.

## Conclusion

We have experimentally investigated valley-momentum-coupled emission in hybrid nanophotonic structures consisting of CVD-grown 1L-WSe_2_ integrated with circular-polarization-selective directional nanoantennas. The bare nanoantenna arrays exhibit an antisymmetric angular CD of about 6% in white light scattering, whereas the corresponding hybrid structures show reduced values of about 2% in valley-selective photoluminescence. Crucially, these effects were observed without the use of polarization analyzers, unambiguously highlighting the central role of angle-selective coupling between the nanoantennas and the emission from valley-polarized exciton populations. Further, we have discussed the experimental conditions required to distinguish genuine valley-momentum locking from farfield polarization-dependent directionality, an essential consideration for leveraging nanophotonic concepts in valleytronic applications. These experimental findings are supported by a reciprocity-based numerical framework that enables modeling of incoherent, valley-selective emitters in periodic nanostructures. The simulations reproduce the experimentally observed reduction of the angular CD in nanoantenna arrays homogeneously covered by a monolayer compared to the response predicted for a single nanoantenna and a single emitter. By systematically varying the monolayer coverage within a single unitcell, we showed that this discrepancy arises from the diminished coupling of emitters located farther from to the nanoantenna. In contrast, angular CD magnitudes exceeding 50% are preserved in simulations when the monolayer is confined to the nanoantenna gap region. Importantly, such spatial confinement is experimentally feasible using established nanopatterning techniques for monolayer TMDs.^73,74^ Finally, we demonstrated that the resulting directional intensity contrast is inherently robust due to the extrinsically chiral nature of the dimer nanoantenna. This robustness establishes resonant nanoantenna architectures as a promising platform for valleytronic processing based on tailored valley-momentum coupling.

Generally, the performance of nanoscopic valleytronic devices based on the nonvanishing DOVP will be challenged by two aspects: (1) the efficient optical addressing and reading out the valley degree of freedom in the presence of resonant nanostructures due to complex nearfield effects both at the excitation and emission level. Importantly, our scheme for valley-routing does not depend on the polarization state of emission in the farfield but relies on the valley-selective nearfield excitation alone. This provides a practical solution to overcome the limitation of an otherwise partly or fully obscured valley-information as measured in the farfield by encoding the valley-information in the PL emission direction. (2) Operating those devices at room temperature due to ultrafast spin-relaxation processes. Promisingly, several works demonstrate a nonzero DOVP at room temperature involving charge capturing,^75^ alloying,^76^ multilayer^14^ and heterostructure^77^ materials paving the way for room temperature plasmonic and dielectric nanoantenna based valleytronic devices.

## Methods

### Fabrication and Nearfield Characterization of Gold Nanoantennas

Gold nanoantennas were fabricated on a thermally oxidized silicon wafer (300 nm silicon dioxide) using electron-beam lithography followed by a lift-off process. Initially, the electron-beam resist (ZEP520A) was spin-coated on the precleaned silicon wafer at 4000 rpm for 40 s and baked at 180 °C for 120 s. Next, the nanoantenna arrays were defined by electron-beam lithography using a base dose of 100 μC cm^–2^ and dose factor variations between 1.2–1.6 and 1.4–1.8 for the large and small nanobars, respectively. After electron beam exposure, the resist was developed in N-amyl acetate for 90 s and rinsed by isopropanol to remove the exposed areas of the resist. Subsequently, 40 nm of gold were deposited using electron-beam evaporation (>99.5% target purity, 9 × 10^–6^ Torr chamber pressure). For lift-off, the coated substrate was left for several hours in a N–N dimethylacetamide bath followed by 10 s ultrasonication and rinsing with acetone and isopropanol.

Cathodoluminescence imaging was performed using a FEI Verios scanning electron microscope equipped with a Gatan MonoCL4 Elite detection system. The region of interest was raster-scanned with a focused electron beam at a magnification of 72000, using an acceleration voltage of 30 keV, a lateral step size of 8 nm, and a dwell time of 0.5 s per pixel. To mitigate beam-induced charging and sample drift during the long dwell-time acquisition, the nanoantenna arrays were fabricated on a substrate with a 10 nm indium tin oxide capping layer. In addition, active drift correction was applied throughout the measurement.

### Optical Experiments

Angle-resolved white light spectroscopy was performed using a stabilized tungsten-halogen light source. The white light was prepared under circular polarization using a linear polarizer and a superachromatic quarter wave plate and focused onto the sample by a 100*x*/0.88NA objective resulting in an illumination spot diameter of about 5.5 μm. In reflection geometry, the backscattered light was collected by the same objective, passed through a 30­(R):70­(T) plate beam splitter, and analyzed in wavelength-momentum space by imaging the back-focal plane of the objective onto the entrance slit of an imaging spectrometer (Andor Shamrock 750). This resulted in a spectral and momentum resolution of Δλ = 0.23 nm and Δ*k*_*x*_/*k*_0_ = 0.016. The momentum-axis was defined by orienting the sample with respect to the orientation of the spectrometer slit. A sketch of the optical setup and a detailed discussion of the polarization control are provided in Section S.1 of the Supporting Information.

Room temperature photoluminescence measurements were conducted using a commercial confocal fluorescence lifetime imaging microscope (MicroTime 200, PicoQuant). A pulsed laser source (100 ps pulse length, 40 MHz repetition rate, 30 μW average power) at 532 nm wavelength was used for excitation and focused on the sample by a 100*x*/0.95NA objective resulting in an estimated spot diameter of 2*r* = 2λ/(NA·π) ≈ 0.36 μm. The signal was collected by the same objective in reflection geometry. The reflected laser light was blocked using a 550 nm long-pass filter.

Polarization-resolved cryogenic photoluminescence measurements were conducted at a temperature of 3.8 K using a closed-loop helium cryostat (s50, Montana Instruments). A 633 nm wavelength continuous-wave helium-neon laser (100 μW average power) was used for excitation. The laser light was prepared under circular polarization using a linear polarizer and a quarter wave plate and focused on the sample by a 100*x*/0.88NA objective resulting in an estimated spot diameter of 2*r* = 2λ/(NA·π) ≈ 0.46 μm. The signal was collected in reflection geometry by the same objective, passed through a dichroic beam splitter and analyzed in a helical polarization basis by a superachromatic quarter wave plate and a linear polarizer. For angle-resolved PL imaging, the back-focal plane of the objective was directly imaged onto an electron-multiplying charge-coupled device (EMCCD, iXon897 Ultra, Andor) or onto the entrance slit of an imaging spectrometer as described above. On the EMCCD, this resulted in a momentum resolution of Δ*k*_*x*_/*k*_0_ = 0.011. A sketch of the optical setup and a detailed discussion of the influence of the optical components on the detection polarization are provided in Section S.1 of the Supporting Information.

### Numerical Simulations

For modeling incoherent emission from periodic nanostructures, we employ an Averaged Reciprocal Modal Analysis (ARMA), exploiting Lorentz reciprocity. This allows us to infer the emission pattern of a two-dimensional semiconductor from its plane wave excitation response.^67,68^

We model this by scanning a plane wave over a regular grid of incident wavevectors **k** = (*k*_*x*_, *k*_*y*_), bound by the numerical aperture |*k*| ≤ *NA* = 0.9, and computing the respective nearfield intensity distribution of the nanoantenna array at the emission wavelength and within the monolayer plane. By reciprocity, we approximate the polarization-dependent angular PL intensities measured in the experiment by the obtained spatially averaged nearfield intensity.

Applying this concept to valley-selective emission, we additionally identify the circularly polarized components of the nearfield to emission contributions obtained from respective valley-excitons. Ultimately, we obtain a polarization-dependent spatially averaged nearfield intensity in *k*-space, allowing qualitative analysis of emission directionality features connected to polarization and nearfield asymmetries.

### Fourier Modal Method

For the full-wave calculations, we employed a custom three-dimensional Fourier modal method (also known as rigorous coupled-wave analysis) with two-dimensional in-plane periodicity.^78–80^ The simulation domain is a 1 μm × 1 μm unitcell containing the nanobar dimer and the layered dielectric stack (compare Figures 1 and 2). The angular space of the incident plane waves is defined in *k*-space via

1 $$\begin{array}{ll} k_{x} & =\sin\left(\theta \right)\cos\left(\varphi \right)\\ k_{y} & =\sin\left(\theta \right)\sin\left(\varphi \right) \end{array},\Rightarrow ,\begin{array}{ll} \theta & =\arcsin\left(\sqrt{k_{x}^{2}+k_{y}^{2}}\right),\\ \varphi & =\arctan2\left(k_{y},k_{x}\right), \end{array}$$

where $\sqrt{k_{x}^{2}+k_{y}^{2}}\leq 0.9$ . The numerical solutions obtained by the Fourier modal approach yield the scattering matrix of the layered system, whose entries are the complex amplitudes of all reflected and transmitted diffraction orders at the monolayer plane. The evanescent mode coefficients then comprise the nearfield components originating from the nanoantenna that interact with the adjacent layers.^72,81^

The electric field components in a linear polarization basis then follow from the Rayleigh-expansion,^78^ as

2 $$E_{n}\left(x,y,z\right)=\sum_{l,m=-N}^{N}E_{n,lm}\exp\left(i\left[\gamma_{lm}z+k_{x,l}x+k_{y,m}y\right]\right)$$

where $E_{n,lm}$ are the Rayleigh coefficients of the *n* – th field component, with *n* ∈ {*x*, *y*, *z*}, obtained from the scattering matrix coefficients (*l*, *m*). The propagation constant in *z*-direction is given by $\gamma_{lm}=\sqrt{{\left(ñk_{0}\right)}^{2}-k_{x,l}^{2}-k_{y,m}^{2}}$ , with the vacuum wavenumber *k*_0_ and the refractive index of the surrounding medium *ñ*.

### Helical Basis Projection

To impose circular polarization, we first project the incident field from its *s*-*p* basis^78^ in spherical coordinates onto the in-plane Cartesian coordinate system of the monolayer. For a given emission direction **k** = (sin θ cos φ, sin θ sin φ, cos θ), we find

3 p=(cos⁡θcos⁡φ,cos⁡θsin⁡φ,−sin⁡θ)

4 s=(−sin⁡φ,cos⁡φ,0)

with the respective transformation matrix

5 $$U=\left[\begin{array}{l} p\\ s \end{array}\right]\cdot \left[xy\right]=\left(\begin{array}{ll} \cos\theta \;\cos\varphi & \cos\theta \;\sin\varphi \\ -\sin\varphi & \cos\varphi \end{array}\right)$$

For oblique emission angles, any circularly polarized farfield component yields an elliptical projection onto the monolayer plane, with the ellipticity and orientation of the polarization ellipse being determined by the emission angle. Hence, we find the in-plane nearfield distribution ${\left(E_{\pm ,x}^{\prime },E_{\pm ,y}^{\prime }\right)}^{T}$ upon a σ ^±^ polarized plane wave, as

6 $$\left(\begin{array}{l} E_{\pm ,x}^{\prime }\\ E_{\pm ,y}^{\prime } \end{array}\right)=\frac{1}{\sqrt{2}}U\left(\begin{array}{l} E_{x}\\ E_{y} \end{array}\right)=\frac{1}{\sqrt{2}}\left(\begin{array}{l} \cos\theta \cos\varphi \cdot E_{x}+\cos\theta \sin\varphi \cdot E_{y}\\ -\sin\varphi \cdot E_{x}+\cos\varphi \cdot E_{y} \end{array}\right)$$

Finally, we transform the obtained in-plane nearfield distribution from a linear polarization basis to a helical basis,^81^ as

7 $$\left(\begin{array}{l} E_{+,\pm }\\ E_{-,\pm } \end{array}\right)=\frac{1}{\sqrt{2}}\left(\begin{array}{ll} 1 & 1\\ i & -1 \end{array}\right)\left(\begin{array}{l} E_{\pm ,x}^{\prime }\\ E_{\pm ,y}^{\prime } \end{array}\right)$$

with the Jones notation σ_farfield_^+^|σ_nearfield_^–^≡+–. In this helical bases, we are able to compare the emerging patterns in *k*-space from the ARMA approach to the measured valley-selective angular PL intensities.

### Field Averaging and Box-Size Dependence

In order to obtain the contribution of the nearfield intensities, reciprocally yielding emission contributions in different angular directions, we calculate the averaged nearfield intensity related to that plane wave direction

8 $$M^{\pm }\left(k_{x},k_{y};\left\{x,y\in box\right\}\right)=\frac{1}{N_{box}}\sum_{\left\{x,y\in box\right\}}|E_{\sigma^{\pm }}\left(k_{x},k_{y};x,y\right){|}^{2}$$

We mimic the experimental conditions by choosing an integration box covering the entire unitcell area.

Additionally, by varying the averaging box size, we are able to investigate both spatially- and polarization-dependent directional coupling effects between valley-selective emitters and the nanobar dimer antennas.

## Supplementary Material



## Notes

Sketch of the optical setup and characterization of the polarizing properties of the optical components. (Sec. S.2) diffractive grating orders and grating order dispersion in momentum-space. (Sec. S.3) Room temperature photoluminescence characterization of 1L-WSe_2_ placed on top of a fabricated gold nanoantenna array. (Sec. S.4) spectral analysis of the excitonic PL from 1L-WSe_2_. (Sec. S.5) comparison of numerical and experimental CD values. (Sec. S.6) Numerically calculated angular emission patterns. (Sec. S.7) symmetry properties and extrinsic chirality of the nanoantenna array. (Sec. S.8) angle-integrated farfield intensity contrast. (Sec. S.9) Farfield circular polarization contrast (PDF)

## Notes

A preprint version of this work is available as Tobias Bucher; Jingshi Yan; Jan Sperrhake; Zlata Fedorova; Mostafa Abasifard; Rajeshkumar Mupparapu; Haitao Chen; Emad Najafidehaghani; Khosro Zangeneh Kamali; Antony George; Mohsen Rahmani; Thomas Pertsch; Andrey Turchanin; Dragomir N. Neshev; Isabelle Staude. Valley-dependent emission patterns enabled by plasmonic nanoantennas. 2025, arXiv:2509.21023. https://arxiv.org/abs/2509.21023 (accessed March 10, 2026).
