# Supplementary material for: Valley-Dependent Emission Patterns Enabled by Plasmonic Nanoantennas
Source: ACS Nano. 2026 Mar 24;20(14):10845–57. doi: 10.1021/acsnano.5c11672 (PMC13085853; doi:10.1021/acsnano.5c11672)
Supplement: Supplementary file 1 [file nn5c11672_si_001.pdf]

Supporting Information: Valley-dependent emission patterns enabled by plasmonic nanoantennas

Tobias Bucher,[*,†,‡,¶] Jingshi Yan,[§] Jan Sperrhake,[‡,¶] Zlata Fedorova,[†,‡,¶] Mostafa Abasifard,[†,‡,¶] Rajeshkumar Mupparapu,[‡,¶] Haitao Chen,[§] Emad Najafidehaghani,[‖] Khosro Zangeneh Kamali,[§] Antony George,[‖,¶] Mohsen Rahmani,[§] Thomas Pertsch,[‡,¶,⊥,#] Andrey Turchanin,[‖,¶,@] Dragomir N. Neshev,[△] and Isabelle Staude[†,‡,¶]

†*Institute of Solid-State Physics, Friedrich Schiller University Jena, 07743 Jena, Germany*

‡*Institute of Applied Physics, Friedrich Schiller University Jena, 07745 Jena, Germany*

¶*Abbe Center of Photonics, Friedrich Schiller University Jena, 07745 Jena, Germany*

§*ARC Centre of Excellence for Transformative Meta-Optical Systems (TMOS), Research School of Physics, Australian National University, Canberra ACT 2601, Australia*

‖*Institute of Physical Chemistry, Friedrich Schiller University Jena, 07743 Jena, Germany*

⊥*Fraunhofer Institute for Applied Optics and Precision Engineering IOF, 07745 Jena, Germany*

#*Max Planck School of Photonics, Germany*

@*Jena Center for Soft Matter (JCSM), 07743 Jena, Germany*

△*ARC Centre of Excellence for Transformative Meta-Optical Systems (TMOS), Research School of Physics, Australian National University, Canberra ACT 2600, Australia*

E-mail: tobias.bucher@uni-jena.de

S.1 Influence of the optical components on the polarization state of light

We performed optical experiments in reflection mode as illustrated in Figure S1a and Figure S2a. In these configurations, the incoming light is initially reflected by a beam splitter (see position (1)) and focused onto the sample inside the cryostat via an objective lens. The circular polarization state of the excitation/illumination is routinely checked before entering the objective. The light emitted from the sample is collected by the same objective, transmitted through the beam splitter (see position (2)), and redirected by a mirror (see position (3)). In the following, we examine the influence of the beam splitter and the mirror on the measured polarization state of the light collected in this configuration. For this, we use the Stokes-formalism of describing (partially) polarized light. The normalized Stokes vector $\mathbf{S} = (1, S_1, S_2, S_3)$ is then defined as follows:

$$S_1 = \frac{I_\mathrm{s} - I_\mathrm{p}}{I_\mathrm{s} + I_\mathrm{p}}, \quad S_2 = \frac{I_\mathrm{d} - I_\mathrm{a}}{I_\mathrm{d} + I_\mathrm{a}}, \quad S_3 = \frac{I_{\sigma^+} - I_{\sigma^-}}{I_{\sigma^+} + I_{\sigma^-}}, \tag{1}$$

where I denotes the light intensities measured for different polarization configurations in detection, as indicated by their subscripts: linear vertical (s) and horizontal (p), linear diagonal (d) and antidiagonal (a), as well as left-handed (σ^+) and right-handed (σ^-) circular polarization.

S.1.1 Circular polarization resolved measurements

S.1.1.1 Scheme for polarization control

For circular-polarization resolved measurements (see Fig. 4(b,c) and Fig. 5 in the main text) we have used a longpass dichroic beam splitter (DBS) with a cut-on wavelength of 650 nm as shown in Figure S1a. As DBS are known to change the polarization state of elliptically polarized light, we have employed the following scheme: We used a combination

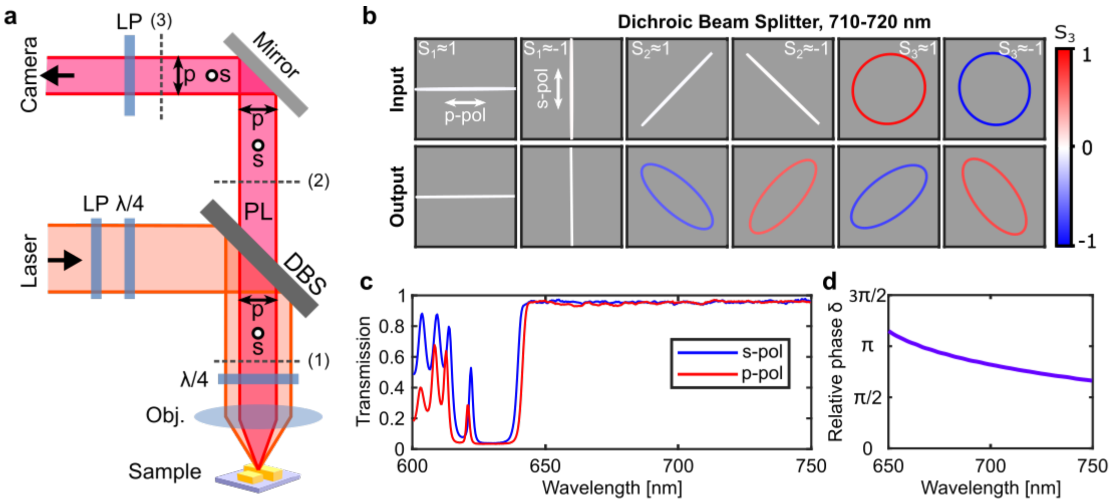

Fig. S1: Optical setup and polarization control. (a) Sketch of the experimental setup. The numbers show different positions of the setup where the polarization state can be examined. (b) The effect of the dichroic beam splitter on the polarization state of light in transmission. The upper row of the table shows the polarization ellipses of the incoming light. The bottom row are the corresponding polarization ellipses being modified by the DBS normalized to the incoming intensity. The measurements are averaged over the spectral range from 710 nm to 720 nm, the colorcode of the ellipses represents their S_3 value. (c) Wavelength-dependent transmission of the DBS for s- and p- polarized light. (d) The relative phase shift between s- and p- components induced by the DBS.

of a linear polarizer (LP) and a quarter-wave plate (QWP) as compensating elements to prepare s-polarized light after the reflection from the DBS (position (1)). The s-polarized light was then sent through a super-achromatic QWP in order to prepare $\sigma^{\pm}$ polarized light before entering the objective. In detection, the same super-achromatic QWP is used to convert the circular polarized components of the collected light into a linear basis and the s- and p-polarized light is transmitted through the DBS (position (2)), reflected by a mirror (position (3)) and analyzed by a LP.

S.1.1.2 Dichroic beam splitter

We examined the polarizing effect of the DBS on the transmitted light in a custom-built white-light spectroscopy setup designed for near-zeroth order transmittance (NA$\approx$ 0.044) at a 45° incidence angle. In this configuration, incoming white light, prepared in an arbitrary

polarization state, passes through the DBS and is fiber-coupled to a spectrometer. A QWP in a motorized rotation mount and a fixed LP are positioned before the spectrometer fiber to measure the Stokes vector components of the transmitted light using the Fourier method. We characterized the transmitted light across six degenerate input polarization states as defined for the Stokes parameters above. Figure S1b shows the polarization ellipses of the incident (upper row) and transmitted (lower row) light averaged over a narrow spectral range of 710 nm to 720 nm. We observe that: (1) s- and p-polarized states remain almost unchanged, (2) diagonal linear polarization turns into elliptical, while the circular states become elliptical as well and their S_3 parameter changes sign. From these observations we can conclude that the DBS induces a phase shift δ between s- and p-polarized field components.

Figure S1c and Figure S1d show the measured transmittance and relative phase shift spectra of the DBS, respectively, where $T_{s/p} = I^{\text{out}}_{\text{s/p}}/I^{\text{in}}_{\text{s/p}}$ and the relative phase shift δ between s- and p-polarized components was extracted from analyzing how the diagonal and circular-polarized components were modified by the DBS, namely, $\delta = \phi^{\text{in}} - \phi^{\text{out}}$, where $\phi^{\text{in/out}} = \tan^{-1}\left(\frac{S_3^{\text{in/out}}}{S_2^{\text{in/out}}}\right)$. The phase shift was extracted as an average over four measurement sets (two diagonal and two circular states were analysed) with a standard deviation of < 0.012 rad. For the wavelength range from 690 nm to 750 nm we find that indeed the amplitude modulation of the DBS is negligible ($T_s \approx T_p \approx 0.96 = T$) but a significant phase shift is introduced.

Next, to characterize the polarization behavior of the DBS, we utilize the Müller matrix formalism. This approach relates the output Stokes vector $\mathbf{S}^{\text{out}}$ to the input Stokes vector $\mathbf{S}^{\text{in}}$ by means of a 4x4 matrix $\hat{\text{M}}_{\text{DBS}}$ such that $\mathbf{S}^{\text{out}} = \hat{\text{M}}_{\text{DBS}}\mathbf{S}^{in}$. Given our observations, the

DBS acts as a waveplate and consequently, its Müller matrix takes the following form:

$$\hat{\mathrm{M}}_{\mathrm{DBS}}(\lambda) = T \begin{bmatrix} 1 & 0 & 0 & 0 \\ 0 & 1 & 0 & 0 \\ 0 & 0 & \cos\delta(\lambda) & -\sin\delta(\lambda) \\ 0 & 0 & \sin\delta(\lambda) & \cos\delta(\lambda) \end{bmatrix}. \quad (2)$$

S.1.1.3 Mirror

After the DBS, the light is reflected from a protected silver mirror under 45°. The Müller matrix for reflection from metals can be given in terms of the complex reflection coefficients r_s and r_p

$$M_{\mathrm{Mirror}} = \frac{1}{2} \begin{bmatrix} r_s^2 + r_p^2 & r_s^2 - r_p^2 & 0 & 0 \\ r_s^2 - r_p^2 & r_s^2 + r_p^2 & 0 & 0 \\ 0 & 0 & 2r_s r_p \cos\gamma & -2r_s r_p \sin\gamma \\ 0 & 0 & 2r_s r_p \sin\gamma & 2r_s r_p \cos\gamma \end{bmatrix}, \quad (3)$$

where $\gamma = \phi_s - \phi_p$ is the phase offset between s- and p- polarized field components. We assume that this phase shift is approximately constant through the examined wavelength range. According to characterization by the manufacturer (Thorlabs), the reflectance of the protected silver mirror at 710 nm wavelength and for 45° incidence angle are $r_s^2 = R_s \approx 0.96$ and $r_p^2 = R_p \approx 0.95$. Hence, we approximate that $R_s \approx R_p \equiv R$ with reasonable accuracy.

S.1.2 Angle-resolved spectroscopy

S.1.2.1 Scheme for polarization control

For angle-resolved spectroscopy measurements (see Fig. 2(d) and Fig. 4(d-g) in the main text) we have used the setup configuration as shown in Figure S2a with a 30(R):70(T) plate beam splitter (BS). As plate BS are known to change the polarization state of elliptically polarized light, we have employed the following scheme: We used a combination of a LP and

a QWP as compensating elements to prepare circular polarized light after the reflection from the BS (position (1))and before entering the objective. In detection, the circular polarized components of the collected light are transmitted through the BS (position (2)), reflected by a mirror (position (3)) and analyzed by a super-achromatic QWP and a LP.

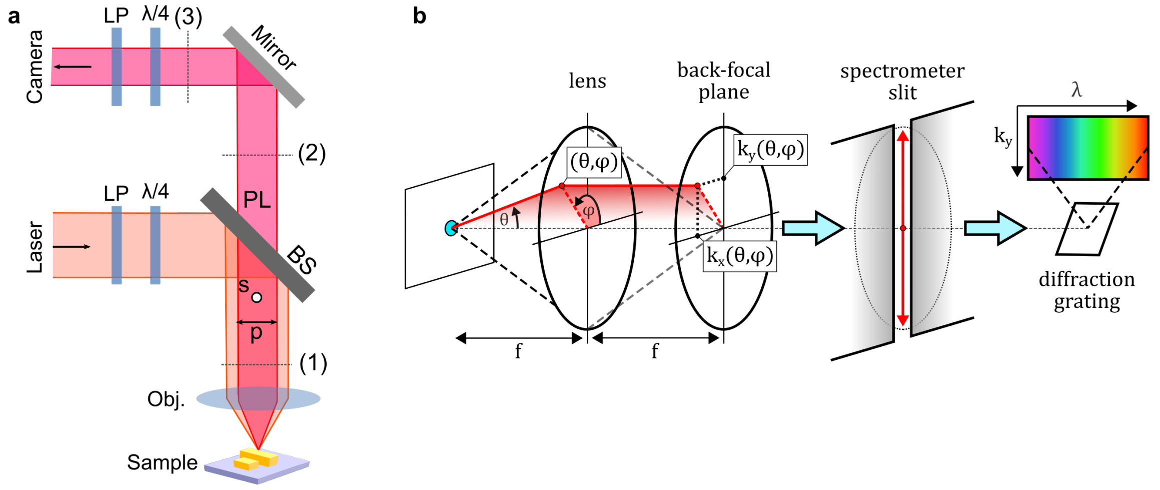

Fig. S2: Angle-resolved spectroscopy setup. (a) Sketch of the experimental setup configuration used for angle-resolved spectroscopy. (b) Sketch of the imaging setup used for angle-resolved spectroscopy.

S.1.2.2 30(T):70(R) plate beam splitter

We have provided a detailed characterization of the polarizing properties of the BS in Sec. S.1 of the Supporting Information of a previous work.[1] In short, the 30(T):70(R) beam splitter acts as a linear polarizing element with anisotropic amplitude attenuation coefficients p_s and p_p, where $T_s = p_s^2$ and $T_p = p_p^2$ are the transmittances for s- and p-polarization, respectively. The measured transmittance spectra of the beam splitter showed a minute wavelength dependence and an average transmittance of $T_s = 0.86$ and $T_p = 0.61$ in the wavelength range of 650 nm to 750 nm. If we consider a partially circular-polarized input state, represented by its Stokes vector, $\mathbf{S}^{\mathrm{in}} = [1, 0, 0, \alpha]^{\mathrm{T}}$ where $\alpha \in (-1, 1)$, the light transmitted through the beam splitter would be described by $\mathbf{S}^{\mathrm{out}} = \hat{\mathrm{M}}_{\mathrm{BS}}\mathbf{S}^{in} = 0.735 \cdot [1, 0.170, 0, 0.985 \cdot \alpha]^{\mathrm{T}}$ as previously shown. Therefore, the degree of circular polarization (DOCP) or S_3 of the light

collected by the objective is slightly decreased by a factor of 0.985 after passing through the beam splitter which is comparable to the natural fluctuations occurring for measurements upon repetition or different sample positions.

S.1.2.3 Back-focal plane imaging

In order to resolve the collected light in angular-space, we have imaged the back-focal plane (BFP) of the objective onto the entrance slit of an imaging spectrometer (Shamrock 750, Andor) as sketched in Figure S2b. We have used two consecutive 4f imaging arms in order to image the BFP of the objective onto the spectrometer slit such that $k = 0$ is falling onto the center of the slit. By closing the spectrometer slit ($\approx 150\,\mu\text{m}$), we isolate a narrow slice of the BFP (including $k = 0$) whose orientation is defined by rotating the sample. The slice of the BFP which is transmitted through the slit is then collected by a parabolic silver mirror inside the spectrometer, sent onto a 1D diffraction grating (150 lmm^{-}1) and imaged onto a charge-coupled device (CCD) camera (iDus420, Andor). This resulted in a spectral and momentum resolution of $\Delta\lambda = 1.4\,\text{nm}$ and $\Delta k_x/k_0 = 0.016$.

S.2 Diffractive grating orders in momentum space

We have analyzed the influence of the diffractive grating orders exhibited by the periodic nanoantenna array (square lattice period $\Lambda = 1\,\mu\text{m}$) in momentum space. Figure S3a shows a sketch of the nanoantenna array and the primitive unit cell of the square lattice (red lines). We then find the respective (reciprocal) lattice vectors, expressed in the xy-coordinate system

(black arrows), as

$$\text{real space:} \quad \mathbf{a}_1 = \frac{\Lambda}{\sqrt{2}}\begin{pmatrix}1\\1\end{pmatrix}, \quad \mathbf{a}_2 = \frac{\Lambda}{\sqrt{2}}\begin{pmatrix}1\\-1\end{pmatrix} \quad \text{and} \tag{4}$$

$$\text{reciprocal space:} \quad \mathbf{b}_1 = \frac{\sqrt{2}\pi}{\Lambda}\begin{pmatrix}1\\1\end{pmatrix}, \quad \mathbf{b}_2 = \frac{\sqrt{2}\pi}{\Lambda}\begin{pmatrix}1\\-1\end{pmatrix}. \tag{5}$$

The linear scattering of a plane wave with incidence angles (θ, ϕ), where $\theta \in [0, \pi]$ is the azimuthal angle measured from the direction of the substrate normal and $\phi \in [0, 2\pi)$ is the polar angle, follows

$$\mathbf{k}'_{||} = \mathbf{k}_{||} + \mathbf{G}_{kl} \ , \quad \text{with} \quad \mathbf{G}_{kl} = k\mathbf{b}_1 + l\mathbf{b}_2 \ , \quad k, l \in \mathbb{Z} \tag{6}$$

where $\mathbf{k}_{||} = k_0(\cos\varphi\sin\theta, \sin\varphi\sin\theta)^{\mathrm{T}}$ and $\mathbf{k}'_{||}$ are the in-plane wavevectors of the incident and scattered wave, respectively, and $k_0 = 2\pi/\lambda$ is the wavenumber at wavelength λ. In the farfield, we only observe scattered waves if their out-of-plane component k'_z is real or equivalently

$$n^2k_z'^2 = n^2k_0^2 - \left(k_0\cos\varphi\sin\theta + (k+l)\frac{\sqrt{2}\pi}{\Lambda}\right)^2 - \left(k_0\sin\varphi\sin\theta + (k-l)\frac{\sqrt{2}\pi}{\Lambda}\right)^2 \geq 0, \tag{7}$$

where n is the refractive index of the medium. For $k'_z = 0$, Equation 7 describes a set of circles in momentum space with radius nk_0 and center points $-\frac{\sqrt{2}\pi}{\Lambda}((k+l), (k-l))$. Figure S3b shows the respective solutions for $k'_z = 0$ in momentum space for an refractive index of $n = 1.28$. We highlighted eight sets of grating orders which overlap with the k-space within the experimental numerical aperture (NA=0.88) by different color and line style. Here, orders with $|k+l| = |k-l|$ relate to modes propagating along the nearest neighbor direction (i.e. along the direction of the (inverse) lattice vectors) and orders with $|k| = |l|$ relate to modes propagating along the second-nearest neighbor direction (i.e. along the $x-$ and

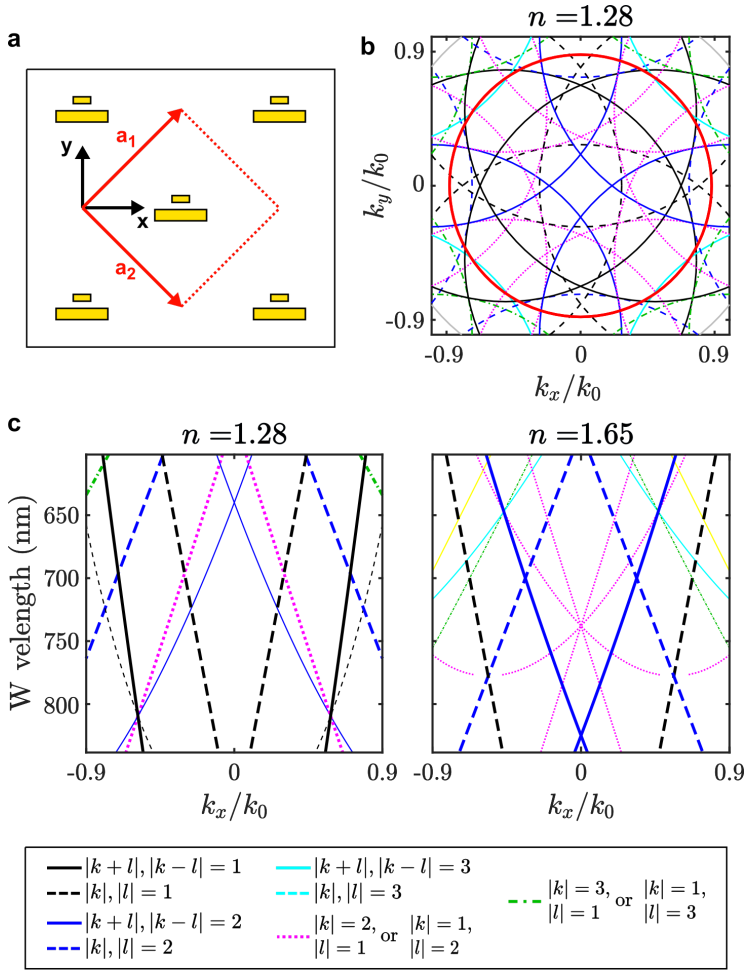

Fig. S3: Diffractive modes of the nanoantenna array. (a) Sketch of the nanoantenna array and the primitive unit cell of the square lattice (red lines). (b) Diffractive grating orders of the square array shown on the left with a lattice constant of $|\mathbf{a}_1| = |\mathbf{a}_2| = 11\,\mu\mathrm{m}$ and a refractive index of $n_1 = 1.28$. The experimental numerical aperture is indicated by a red circle. (c) Grating order dispersion for the same grating as discussed above for $k_y = 0$ considering refractive indices of $n_1 = 1.28$ (left) and $n_2 = 1.65$ (right). In all cases the lowest eight grating orders are highlighted by different colors according to the legend at the bottom.

$y-$direction). Note that we have used the refractive index n as a free parameter to fit the experimentally observed patterns in the back-focal plane images of PL from 1L-WSe_2 on top of the nanoantenna array (see Fig. 5 in the main text). The nanoantennas are embedded in a 15 nm thin layer of silicon dioxide. The low effective refractive index of $n_1 = 1.28$ hints at a non-negligible porosity of the deposited silicon dioxide with pore sizes smaller than the optical wavelength.

By dividing Equation 7 with k_0^2, we can rewrite the grating order equation at $k_x = 0$ (i.e. $\varphi = 0$) to obtain the implicit grating order dispersion relation

$$n^2 - \left(\sin\theta + (k+l)\frac{\lambda}{\sqrt{2}\Lambda}\right)^2 - (k-l)^2\left(\frac{\lambda}{\sqrt{2}\Lambda}\right)^2 \geq 0, \tag{8}$$

where λ is the vacuum wavelength of light. From Equation 8, we find that the orders generally show a quadratic dispersion except for $k = l$ (i.e. the modes exhibited by a 1D grating along x-direction). Figure S3c shows the respective solutions for $k_z' = 0$ in momentum space assuming refractive indices of $n_1 = 1.28$ (left side) for the silicon oxide embedding layer and $n_2 = 1.65$ (right side) for the indium tin oxide capping layer of the substrate. Again, we highlighted eight sets of grating orders which overlap with the k-space within the experimental numerical aperture (NA=0.88) by different color and line style. Note that in Fig. 2d of the main text only those orders are considered which show in the measured momentum-resolved spectra.

S.3 Room temperature photoluminescence

We characterized the hybrid system of monolayer WSe_2 on top of a gold nanoantenna array at room temperature by means of photoluminescence (PL) microscopy and spectroscopy. All measurements at room temperature were performed using a commercial fluorescence lifetime imaging setup (PicoQuant, MicroTime 200). A 530 nm pulsed excitation laser with a 40 MHz repetition rate, 100 ps pulse duration, and $\approx$ 30 µW average power was focused on the sample using a 100x/0.95NA objective resulting in an estimated spot diameter of $2r = 2\lambda/(\mathrm{NA}\cdot\pi) \approx 0.36$ µm. The same objective was used to collect the signal in reflection geometry. Figure S4a shows the measured PL intensity map (longpass 700 nm) presented in Fig. 3b of the main text for logarithmic intensity scaling. Further, Figure S4b shows measured PL intensity spectra from six different positions in the same region of the scan as indicated by circles. The uniformly distributed bright PL signal originates from the pristine

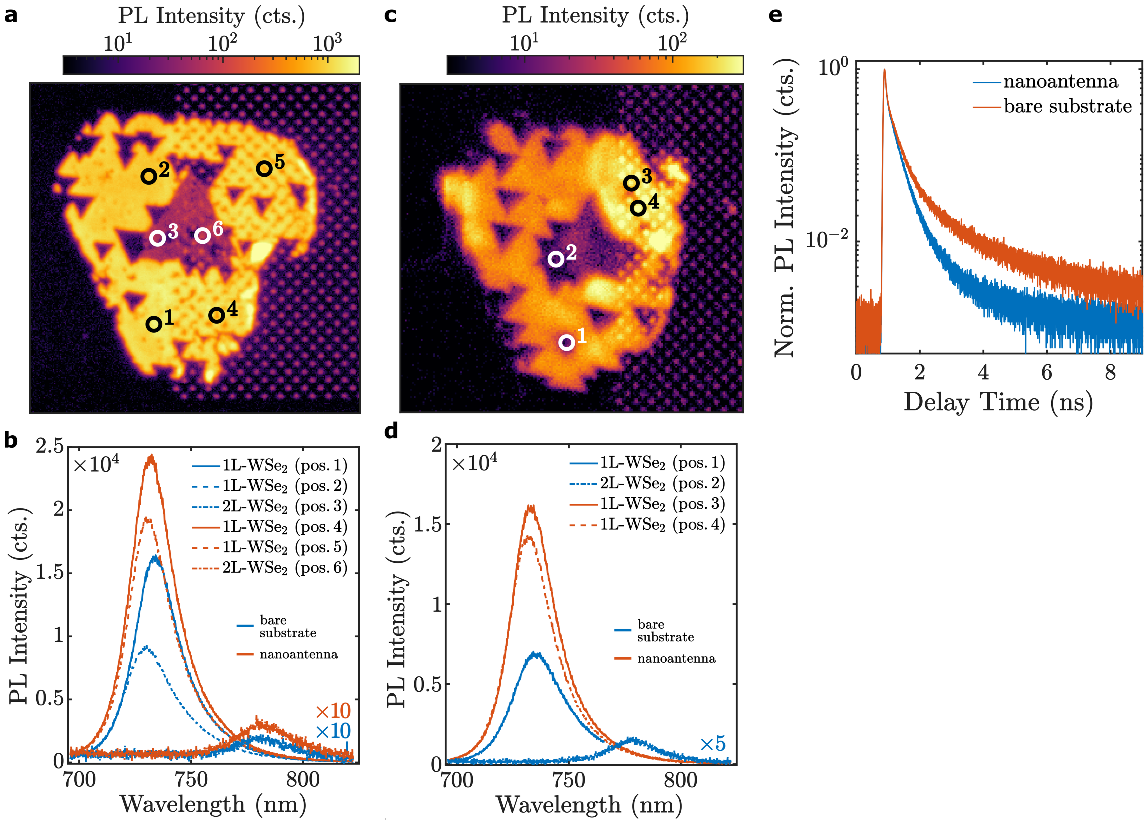

Fig. S4: Additional room temperature PL measurements. (a,c) Measured confocal microscope photoluminescence images from 1L-WSe_2 on top of nanoantenna arrays with different geometrical nanoantenna parameters. (b,d) Respective photoluminescence intensity spectra measured in the positions indicated by circles in (a,c) and labelled by numbers. (e) Measured photoluminescence decay curves of the same WSe_2 crystal as shown in (a) on top of the gold nanoantenna array (orange curve) and on bare substrate (blue curve).

regions of 1L-WSe_2 as confirmed by the PL intensity spectra measured in positions 1, 2, 4, and 5. The respective spectra show a pronounced peak at 730 nm wavelength which is a mixed contribution of the neutral (X) and charged exciton (X^-) in 1L-WSe_2. A large triangular region in the center of the WSe2 crystal shows an about one order of magnitude lower PL intensity and can be attributed to 2L-WSe_2 as seen from the spectrally shifted peak in the PL intensity spectra measured in positions 3 and 6. In several smaller triangular regions no PL signal different from the intrinsic emission of the substrate can be observed. As the visual contrast of these regions is also similar as for the bare substrate (see Fig. 3a of the main text), we conclude that these regions are holes in the WSe_2 crystal.

Further, a regular square pattern of spots is visible in the confocal scan where each spot coincides with the location of an individual nanoantenna. The nanoantenna regions appear relatively brighter on the substrate region without 1L-WSe_2 showing a PL enhancement of the autofluorescence from the substrate due to the nanoantennas. In contrast, the PL from 1L-WSe_2 appears relatively weaker in the position of the nanoantennas which we attribute to the interplay of the resonant nanoantennas with guided modes in the silicon dioxide slab of the substrate. Depending on their geometrical parameters and respective spectral resonance position, the nanoantennas can facilitate an in- or out-coupling from farfield radiation (travelling out of the subtrate plane) to the guided modes in the substrate (travelling in the substrate plane). This interplay leads to a different out-coupling efficiency of PL from emitters depending on their relative position, i.e. in the substrate or on top of the nanoantennas. For comparison, Figure S4c and Figure S4d show the measured PL intensity map and spectra, respectively, for an array with slightly different geometrical nanoantenna parameters. In this case, the relative brightness of PL at the location of the nanoantennas is enhanced further suggesting that the effect of guided modes in the substrate is sensitive to the exact resonant conditions of the nanoantennas.

Interestingly, in all spectral measurements we find an enhanced PL intensity from WSe_2 for the positions of the nanoantennas (orange curves) as compared to bare substrate (blue curves) irrespective of the observations in the confocal scans. Note that the confocal scan were performed with a pixel integration time of 5 ms while the signal for the spectral point measurements was integrated for several 10 s of seconds. This might indicate that the PL response of the WSe_2 on substrate varies over a time scale of milliseconds to seconds which is much slower than electronic processes (picoseconds to nanoseconds). Such effects are typically related to charge-trapping in localized defect states. As a qualitative statement we therefore conclude that the presence of the nanoantennas can enhance the radiative decay rate of defect states in WSe_2. Figure S4e shows a measured decay curve of PL from 1L-WSe_2 on bare substrate (blue curve) and on top of the nanoantenna array (orange curve) from the

same sample as shown in Figure S4a where we have used a 730/10 nm bandpass filter. We observe a two-component decay and by fitting a two-exponential function we extracted PL lifetimes of (2.62 ± 0.05) ns and (0.37 ± 0.01) ns on bare substrate. The decay constants reduce to (1.90 ± 0.06) ns and (0.32 ± 0.01) ns on the nanoantenna array which further shows the presence of a nearfield interaction of the nanoantennas with emitters in 1L-WSe_2.

S.4 Spectral analysis of the excitonic photoluminescence from 1L-WSe_2

We have analyzed the excitonic content of the measured PL spectra from 1L-WSe_2 at cryogenic conditions ($T = 3.8\,K$) using a multi Voigt-line fitting procedure (see for example Bender et al.[2]). For 1L-WSe_2, both in-plane (bright) and out-of-plane (grey/dark) excitons may contribute to the PL emission owing to the negative conduction band splitting in W-based 1L-TMDs.[3,4] In our cryo-PL spectral measurements of 1L-WSe_2 on the bare substrate (see black circles in Figure S5a and blue area in Fig. 4b of the main text), we observe several spectral contributions that we attribute as follows:[5–10] The highest-energy peak corresponds to the neutral bright exciton X^0 (purple area) at a wavelength of 693.8 nm (1.787 eV). The next-highest-energy peak spectrally overlaps with the expected energies of negatively charged trion states, here represented by one contribution X^- (yellow area) at 705.7 nm (1.757 eV), as well as grey/dark exciton states D^0 and trion states D^-, again represented by one contribution X^D (green area) at 708.9 nm (1.749 eV). In the main text, we labeled this peak as X^-/D with its maximum intensity appearing at 706.1 nm (1.756 eV). On the low-energy side of the spectrum, we observe a broad defect-mediated emission band (light blue area) centered at 723.4 nm (1.714 eV) and an almost flat background signal (light pink area) with its center wavelength lying outside of the spectral range of interest. We also observe one additional peak that spectrally overlaps with the expected energy of a localized defect state L_1 and bi-exciton states (XX), here represented by one contribution L_1/XX

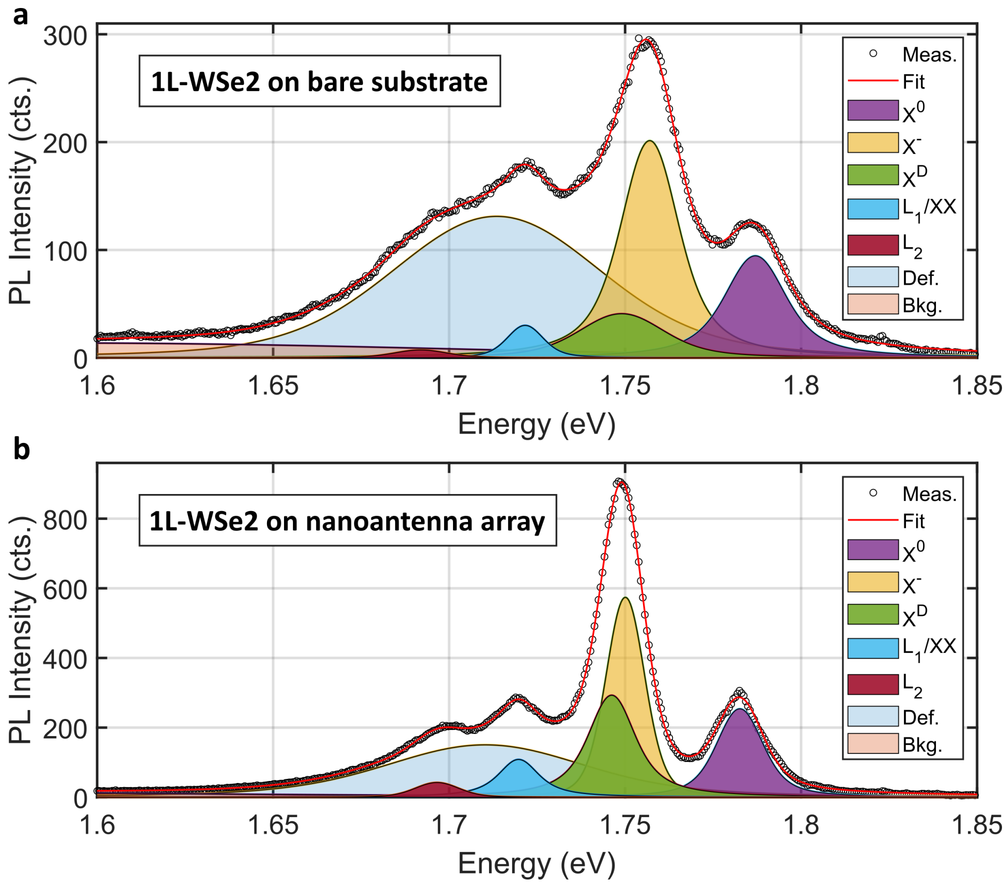

Fig. S5: Voigt-profile spectral analysis. Spectral fitting of the measured cryogenic photoluminescence spectra from 1L-WSe_2 on (a) the bare substrate and (b) the nanoantenna array using σ^+ polarized excitation and detection.

(cyan area) at 720.0 nm (1.722 eV). In the main text, we labeled this peak as L_1/XX with its maximum intensity appearing at 720.4 nm (1.721 eV). We note that the bi-exciton is not expected to be dominant at the relatively low pump fluences used in this work.[5,6] We observe another minute feature attributed to a different localized defect state L_2 (dark red area) at 732.8 nm (1.692 eV). The sum of all contributions (red curve) agrees well with the measured spectrum.

For 1L-WSe_2 on the nanoantenna array (see black circles in Figure S5b and orange area in Fig. 4b of the main text), we observe a similar spectral composition. However, the X^0, X^-/X^D, and L_1/XX peaks are shifted toward smaller energies by 4 meV, 7 meV, and 3 meV, respectively. We attribute the smaller spectral shifts of 3 to 4 meV to natural vari-

ations across the monolayer area and potential modifications in the local strain due to the non-planarized sample surface. Interestingly, for the X^-/X^D peak we observe a larger spectral shift owing to the relatively enhanced dark exciton contribution X^D for 1L-WSe_2 on the nanoantenna array (compare green areas). This enhancement results from coupling to the strong out-of-plane nearfield components mediated by the plasmonic nanoantenna.

Further, we note that we observe a similar DOCP of PL for the X^0 and X^-/D peaks (compare with Fig. 4c of the main text) on both the bare substrate and the nanoantenna array. As the circular polarization contrast of emission is related to valley-polarized in-plane excitons, we conclude that the X^0 and X^-/D peaks exhibit similar in-plane contributions, indicating that the dark exciton contribution X^D is dominated by grey trions.[10]

S.5 Comparison of experimentally and numerically obtained angular circular dichroism maps

We compare the angular circular dichroism (CD) of 1L-WSe_2 on top of the nanobar dimer array obtained by experimental and numerical methods. Figure S6 shows the angular CD along the k_x-direction ($k_y = 0$), obtained at a wavelength of 710 nm by angle-resolved white light (orange markers) and PL (green markers) spectroscopy, angle-resolved PL imaging (blue markers), and numerical emission modeling (red markers). For angle-resolved white light spectroscopy, we find a prominent antisymmetric distribution with respect to the k_x-direction appearing at the angular range marked by the light-gray area. For clarity, we further highlighted the amplitudes of the antisymmetric feature by the dark gray areas. As discussed in the main text, for the angle-resolved PL spectroscopy result, the distribution is less systematic. However, a significant antisymmetric feature with reduced amplitudes appears in the same angular range. Interestingly, the sign of the PL feature is reversed with respect to the white light result. Note that we also observe a sign reversal of the angular

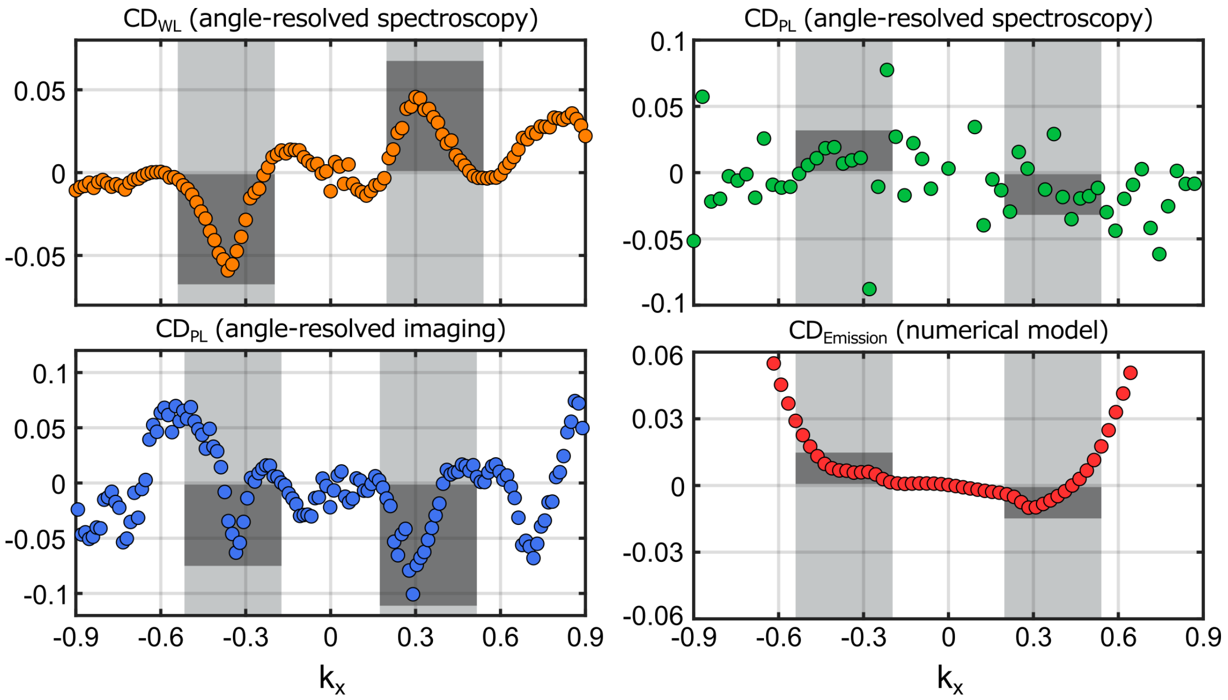

Fig. S6: Comparison of CD distributions. Angular circular dichroism of 1L-WSe_2 on top of the nanobar dimer array along the k_x-direction, obtained at a wavelength of 710 nm by angle-resolved white light (orange markers) and photoluminescence (green markers) spectroscopy, angle-resolved photoluminescence imaging (blue markers), and numerical emission modeling (red markers). The light and dark gray areas were added as a guide to the eye.

CD in PL by our numerical emission model when varying the filling factor of the monolayer (see Fig. 6d of the main text). Hence, the orientation of these antisymmetric features likely depends on the specific nearfield interaction for a given emitter distribution.

In experiments, the whole unitcell of the array is covered with 1L-WSe_2 (100% filling factor). The numerical emission model for this case confirms the angular range of this antisymmetric feature. However, our model predicts a lower amplitude than observed in the angle-resolved spectroscopy measurements. This may indicate that in experiments a stronger nanoantenna response is observed than numerically predicted for a fully covered unitcell, likely caused by local excitation and emission enhancement limited to the close proximity of the nanoantenna.

Ultimately, we compare the angle-resolved PL spectroscopy measurements to our angle-resolved PL imaging results. Note that we limit our discussion to a qualitative comparison,

as both data sets were obtained under different experimental condition: for angle-resolved PL imaging, we employed a 710/10 nm bandpass filter and a tightly focused excitation beam (0.46 µm spot diameter), while for the angle-resolved PL spectroscopy the spectral resolution is determined by the spectrometer (three-pixel spectral width of 0.7 nm) and we employed a widened excitation beam (5.5 µm spot diameter).

The PL imaging result shows larger magnitudes of the CD distribution but no antisymmetric features within the angular range discussed above. We note that pronounced minima appear in the same angular range that relate to diffractive modes. However, their amplitude difference follows the same trend as the antisymmetric features observed for the other PL results.

S.6 Numerically calculated angular emission patterns

Figure S7 shows the numerically calculated angular emission intensities obtained for the nanobar dimer array, as discussed in the main text. For simplicity, we introduce a Jones notation to distinguish between the circular polarization states as follows: $\sigma^{+}_{\text{farfield}} \,|\, \sigma^{-}_{\text{nearfield}} \equiv +-$, with analogous definitions for all other polarization combinations. The calculated emission patterns are in very good agreement with the experimentally obtained angular PL intensities shown in Fig. 5 of the main text. The modeled emission patterns show a clear influence by diffractive modes arising from the periodic arrangement of the nanoantennas within the array. Depending on the circular polarization in the farfield, these diffractive modes appear with different strengths, giving rise to similar polarization-dependent farfield patterns as experimentally observed and discussed in the main text.

S.7 Extrinsic chirality of the nanobar dimer array

In the following discussion, we analyze the geometrical and electromagnetic chiral properties of the nanoantenna arrays, as studied in the main text, and within the context of circularly

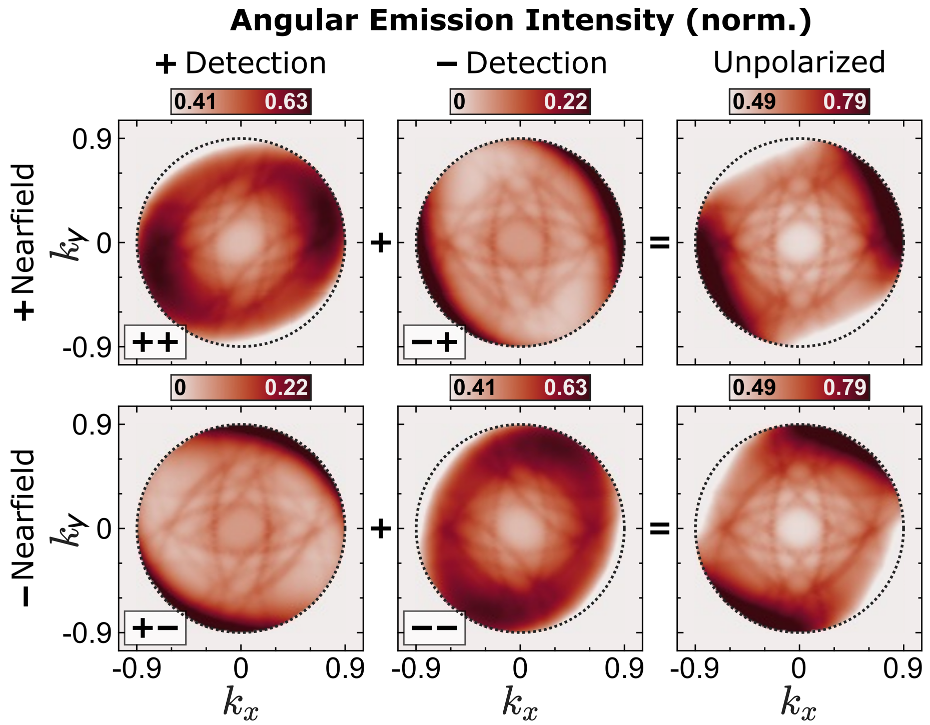

Fig. S7: Emission modeling. Numerically calculated angular emission intensities for the nanobar dimer array, obtained at a wavelength of 710 nm, for circular nearfield polarization (rows) and farfield polarization (left and middle columns) polarizations, as well as for unpolarized farfield (right column).

polarized field polarizations.

True structural chirality requires a lack of any mirror plane in three dimensions,[11] and as Caloz et al. emphasize, "chiral exclusively refers to phenomena in materials composed of particles with structural handedness".[11] In such inherently chiral structures, magnetoelectric coupling (bianisotropy) is the fundamental origin of any optical handedness.[11,12]

A single gold nanobar possesses C_2 symmetry, and a square array of nanobars also retains C_2 symmetry, whenever the nanobar axis aligns with any lattice axis, as shown in Figure S8a. Geometrically, dimers of two non-identical nanobars can retain mirror symmetry (C_S-symmetric) with respect to one axis, as shown in Figure S8b, rendering the dimer achiral in the strict, threedimensional sense.

Along the normal direction (and for small angular deviations), the single nanobar and the nanobar dimer exhibit the aforementioned mirror symmetry in the plane, hence their angular

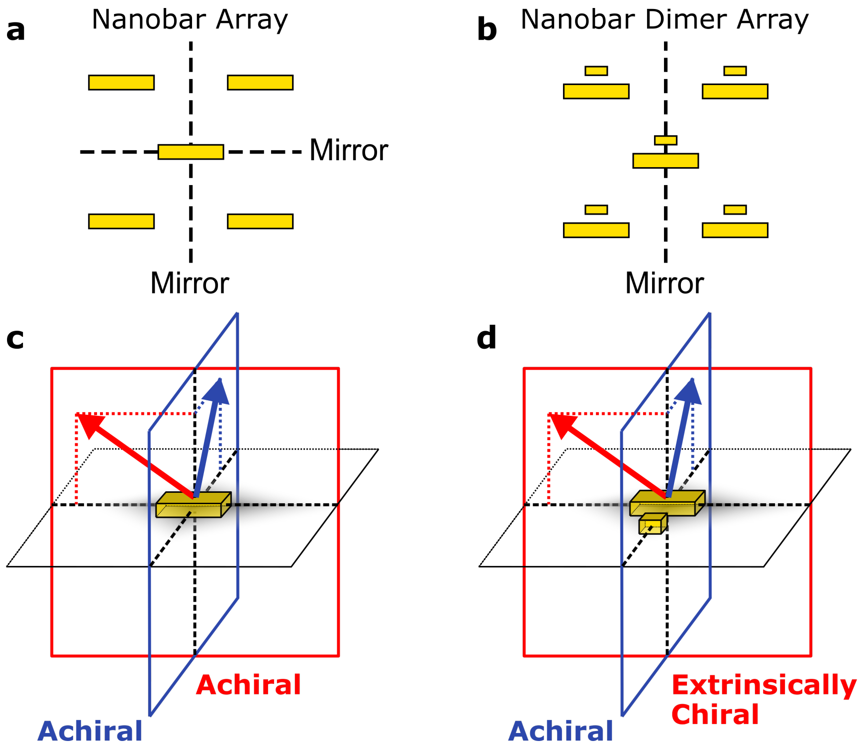

Fig. S8: Symmetry properties and extrinsic chirality. Sketch of an achiral array of (a) single nanobars and (b) nanobar dimers. Symmetry planes and properties for oblique emission angles for the (c) single nanobar array and the (d) nanobar dimer array.

CD must vanish for **k**-vectors near $k_x = k_y = 0$.[13] In contrast, for oblique emission angles the effective symmetry is lowered: each emitted plane wave sees a "tilted" array, and thereby breaking the mirror symmetry with respect to its "plane of incidence".

As discussed above, the single nanobar array and the nanobar dimer array posses different symmetry axes. Hence, we analyze the effect of oblique angles with respect to these axes, as shown in Figure S8c and Figure S8d, respectively. As highlighted by the red and blue arrows, any oblique **k**vector that lies within one of the symmetry planes (red and blue), is breaking the mirror symmetry with respect to the plane normal to its plane of incidence. For the single nanobar array, however, mirror symmetry is always retained due to the second mirror axis, thus rendering the system achiral. In contrast, for the nanobar dimer, an oblique **k**vector that lies within the plane normal to its mirror plane (red arrow), breaks the twodimensional mirror symmetry in threedimensional space. This is the hallmark of *extrinsic* chirality.[13]

Moreover, the observed chiral asymmetry is not generated by the dimer alone but by the entire layered structure. For circularly or elliptically polarized light, the silicon substrate acts as polarizationrotating mirror. Due to the thin spacer it interacts in the near field with the nanobar layer.[14] Furthermore, Plum et al. have shown that, additionally, for planar, achiral resonators on top of a reflector, oblique incidence can produce enhanced chiral effects.[13] In our system, the angular CD signal is most robust when the plane of incidence aligns with the dimer axis, directly linking the handedness of illumination (σ^+ versus σ^-) to directional emission via reciprocity.

S.8 Angle-integrated farfield intensity contrast

The angular emission intensities show a rich behavior when considering different emission angles as well as for different monolayer filling factors. Integrating intensities over many emission angles, hence may significantly lower the intensity contrast. However, as discussed in the main text and in Sec. S.7 of the Supporting Information, the angular asymmetry is robust with respect to the two halfspaces that are linked to extrinsic chirality. Thus, integrating all intensity contributions within each of these halfspaces, should retain a finite intensity contrast. In addition to the fixed-direction intensity contrast provided in Fig. 6 of the main text, we calculated the contrast between these integrated intensities, as shown in Figure S9. The resulting curve clearly shows a finite contrast for the nanobar dimer array. Additionally, the distribution is similar to the result obtained for a fixed farfield direction along the k_x axis (compare Fig. 6d of the main text), as a consequence of the symmetry-protected extrinsic chirality of the two halfspaces.

Importantly, the single nanobar arrays show no significant angle-integrated intensity contrast. This confirms the initial assumption that angle-integration may drastically reduce the observed intensity contrast in the absence of extrinsic chirality.

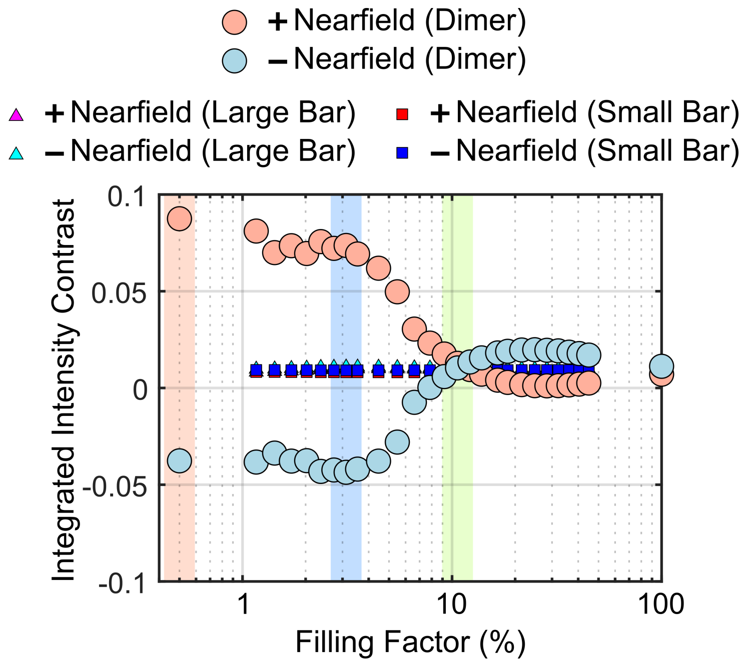

Fig. S9: Angle-integrated farfield intensity contrast. Numerically calculated half-space intensity contrast, obtained from σ^+ and σ^- polarized nearfield components, for the nanobar dimer array (red and blue circles), the large-nanobar array (magenta and cyan triangles), and the small-nanobar array (red and blue squares).

S.9 Farfield circular polarization contrast

Besides valley-selective directional effects, we have also analyzed the farfield circular polarization of the light emitted from the nanoantenna array. For this, we consider the calculated angular emission intensities (e.g. compare Figure S7) and obtain their overall radiated powers P_{++}, P_{-+}, P_{+-}, and P_{--} by integration over all emission angles within the numerical aperture. Figure S10 shows the respective DOCP in the farfield, defined as $\text{DOCP}_{\pm} = (P_{+\pm} - P_{-\pm})/(P_{+\pm} + P_{-\pm})$, obtained for a given nearfield polarization $\pm$ according to the Jones notation introduced in Section S.6. We find that the DOCP gradually decreases with decreasing filling factor, showing that the nanoantenna does not preserve the circular polarization upon farfield scattering. For larger filling factors, the nanoantenna's relative contribution with respect to emission from the whole unitcell, however, becomes less significant, leading to the observed increase in the DOCP. Note that the DOCP without the

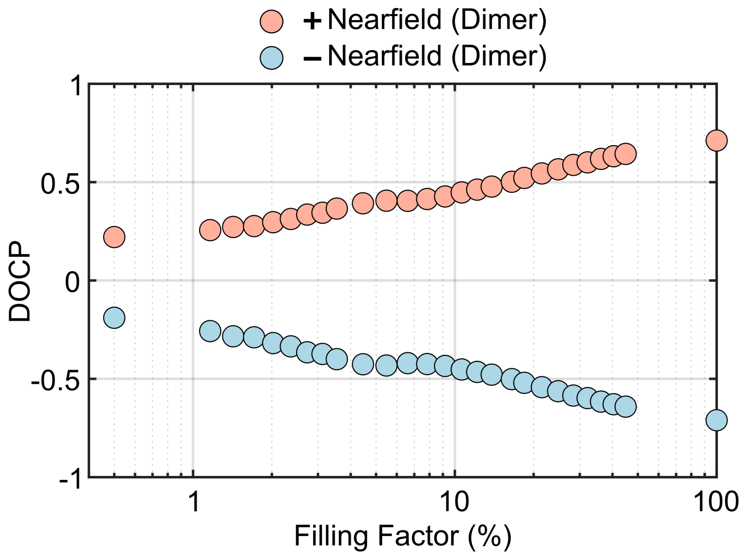

Fig. S10: Farfield circular polarization contrast. Numerically calculated degree of circular polarization of the nanobar dimer array, obtained from the angle-integrated emission patterns for σ^+ and σ^- polarized nearfield components.

nanoantenna, or for an infinitely large unitcell, has a magnitude close to 1.

In conclusion, we find that valley-selective emitters being located within the nearfield region of the nanoantenna, and therefore most strongly interacting with the nanoantenna, exhibit the lowest circular polarization contrast in the farfield. Hence, the valley-information of the localized emitters is obscured in farfield polarization measurements. Importantly, our nanoantenna design allows discerning the valley-information from directional contrast measurements, whose magnitude, in contrast, is larger for smaller filling factors.

References

(1) Bucher, T.; Fedorova, Z.; Abasifard, M.; Mupparapu, R.; Wurdack, M. J.; Najafidehaghani, E.; Gan, Z. Y.; Knopf, H.; George, A.; Eilenberger, F.; Pertsch, T.; Turchanin, A.; Staude, I. Influence of resonant plasmonic nanoparticles on optically accessing the valley degree of freedom in 2D semiconductors. *Nat. Commun.* **2024**, *15*.

(2) Bender, V.; Bucher, T.; Mishuk, M. N.; Xie, Y. X.; Staude, I.; Eilenberger, F.; Busch, K.; Pertsch, T.; Tugchin, B. N. Spectroscopic Study of the Excitonic Structure in Monolayer MoS_2 under Multivariate Physical and Chemical Stimuli. *Physica Status Solidi a-Applications and Materials Science* **2023**,

(3) Kosmider, K.; González, J. W.; Fernández-Rossier, J. Large spin splitting in the conduction band of transition metal dichalcogenide monolayers. *Phys. Rev. B* **2013**, *88*.

(4) Kormányos, A.; Zólyomi, V.; Drummond, N. D.; Burkard, G. Spin-Orbit Coupling, Quantum Dots, and Qubits in Monolayer Transition Metal Dichalcogenides. *Phys. Rev. X* **2014**, *4*.

(5) You, Y. M.; Zhang, X. X.; Berkelbach, T. C.; Hybertsen, M. S.; Reichman, D. R.; Heinz, T. F. Observation of biexcitons in monolayer WSe_2. *Nat. Phys.* **2015**, *11*, 477–U138.

(6) Huang, J. N.; Hoang, T. B.; Mikkelsen, M. H. Probing the origin of excitonic states in monolayer WSe_2. *Sci. Rep.* **2016**, *6*.

(7) Robert, C.; Amand, T.; Cadiz, F.; Lagarde, D.; Courtade, E.; Manca, M.; Taniguchi, T.; Watanabe, K.; Urbaszek, B.; Marie, X. Fine structure and lifetime of dark excitons in transition metal dichalcogenide monolayers. *Phys. Rev. B* **2017**, *96*.

(8) Courtade, E. et al. Charged excitons in monolayer WSe_2: Experiment and theory. *Phys. Rev. B* **2017**, *96*, 085302.

(9) Molas, M. R.; Slobodeniuk, A. O.; Kazimierczuk, T.; Nogajewski, K.; Bartos, M.; Kapuscinski, P.; Oreszczuk, K.; Watanabe, K.; Taniguchi, T.; Faugeras, C.; Kossacki, P.; Basko, D. M.; Potemski, M. Probing and Manipulating Valley Coherence of Dark Excitons in Monolayer WSe_2. *Phys. Rev. Lett.* **2019**, *123*.

(10) Jindal, V.; Mourzidis, K.; Balocchi, A.; Robert, C.; Li, P.; Van Tuan, D.; Lombez, L.; Lagarde, D.; Renucci, P.; Taniguchi, T.; Watanabe, K.; Dery, H.; Marie, X. Brightened emission of dark trions in transition metal dichalcogenide monolayers. *Phys. Rev. B* **2025**, *111*, 155409.

(11) Caloz, C.; Sihvola, A. Electromagnetic Chirality, Part 1: The Microscopic Perspective. *IEEE Antennas Propag. Mag.* **2020**, *62*, 58–71.

(12) Hentschel, M.; Wu, L.; Schaferling, M.; Bai, P.; Li, E. P.; Giessen, H. Optical Properties of Chiral Three-Dimensional Plasmonic Oligomers at the Onset of Charge-Transfer Plasmons. *ACS Nano* **2012**, *6*, 10355–10365.

(13) Plum, E. Extrinsic chirality: Tunable optically active reflectors and perfect absorbers. *Appl. Phys. Lett.* **2016**, *108*, 241905.

(14) Sperrhake, J.; Falkner, M.; Steinert, M.; Fasold, S.; Pertsch, T. Experimental validation of the fundamental mode approximation for stacked metasurfaces and its application to the treatment of arbitrary period ratios. *APL Photonics* **2021**, *6*, 096109.
